# Integrated flexible DNA methylation–chromatin segmentation modeling enhances epigenomic state annotation

**DOI:** 10.1093/nar/gkag591

**Published:** 2026-06-16

**Authors:** Nihit Aggarwal, Johanna Elena Schmitz, Lukas Laufer, Sven Rahmann, Jörn Walter, Abdulrahman Salhab

**Affiliations:** Department of Genetics, Saarland University, 66123 Saarbrücken, Germany; Algorithmic Bioinformatics, Center for Bioinformatics Saar, Saarland Informatics Campus, 66123 Saarbrücken, Germany; Fakultät MI, Saarland University, Saarland Informatics Campus, 66123 Saarbrücken, Germany; Saarbrücken Graduate School of Computer Science, Saarland Informatics Campus, 66123 Saarbrücken, Germany; Department of Genetics, Saarland University, 66123 Saarbrücken, Germany; Algorithmic Bioinformatics, Center for Bioinformatics Saar, Saarland Informatics Campus, 66123 Saarbrücken, Germany; Fakultät MI, Saarland University, Saarland Informatics Campus, 66123 Saarbrücken, Germany; PharmaScienceHub, Saarland University, 66123 Saarbrücken, Germany; Department of Genetics, Saarland University, 66123 Saarbrücken, Germany; Department of Genetics, Saarland University, 66123 Saarbrücken, Germany; Genomics Data Science Core, Integrated Genomics Services, Sidra Medicine, 26999Doha, Qatar

## Abstract

DNA methylation and histone modifications together shape the cell-type-specific epigenomic landscape. To enhance genome-wide annotation, we developed EpiSegMixMeth (ESMM), the first integrative segmentation model combining chromatin marks and DNA methylation. ESMM improves upon hidden Markov models by incorporating flexible read count distributions and state duration modeling. Applied to 154 high-quality human epigenomes from the IHEC EpiATLAS, ESMM enhances the annotation of broad heterochromatic regions—over 60% of the genome—that are often missed by chromatin-only models. It accurately defines narrow regulatory element boundaries and captures local chromatin state transitions during cell differentiation. Notably, we show that DNA methylation can replace missing repressive histone marks in segmentation, ensuring robust results across various cell types. In developing memory B cells, ESMM reveals chromatin shifts that align with 3D genome architecture changes, providing a valuable resource for studying cell-type-specific epigenomic regulation.

## Graphical Abstract

**Graphical Abstract:**
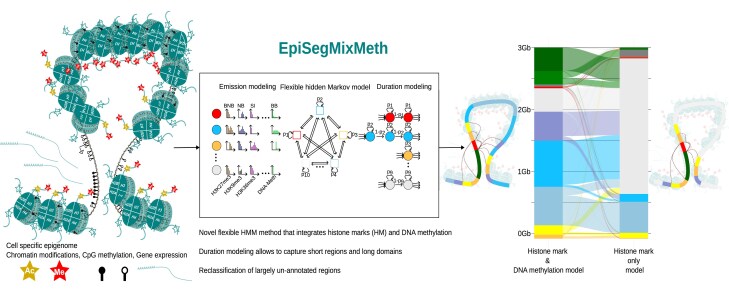

## Introduction

The epigenomic landscape of the genome is comprised by cell-type-specific modifications. A complex combination of DNA methylation and post-translational histone modifications establishes a chromatin *grammar* linked to the regulation of gene expression and the organization of the genome into distinct functional sub-compartments in the nucleus [1–3]. Over the last decade, a number of comprehensive cell-type-specific epigenomes have been collected by national and international consortia. These are currently assembled in a first comprehensive human epigenome cell atlas by the International Human Epigenome Consortium (IHEC) [4,5]. This data is essential to understand the cell type and cell state specific programs executed from a unique genome. Currently, a comprehensive epigenome is defined by DNA methylation, RNA-seq, and (a minimum of) six core post-translational histone modifications that establish the cell-type-specific genome programs [1,5].

Epigenetic modifications operate at different scales, ranging from individual base pairs (DNA methylation) to nucleosomes up to large chromatin domains. There is a significant overlap and crosstalk between these marks [6,7]. For instance, high cytosine methylation correlates with H3K9 methylation [8, 9], while it anticorrelates with the regulatory mark H3K4me3 and, in regulatory regions, additionally with the polycomb-associated repressive mark H3K27me3 [10–14].

Current segmentation and genome annotation (SAGA) algorithms computationally model these complex relationships [15] as a multidimensional linear sequence of epigenetic states and link them to functionally annotated genomic regions, such as promoters, enhancers, and transcriptional states [16–18]. In other approaches, the three-dimensional organization of genomes derived from Hi-C data has been transformed into linear segments (compartments) and linked to chromatin states to better understand the role of the spatial nuclear (chromatin) context, generating a three-dimensional view on the functional organization of gene regulation and chromatin crosstalk [19–21]. All widely used segmentation tools [15] either exclusively use histone marks as input, such as ChromHMM [18], Segway [22, 23], EpiCSeg [17], and EpiSegMix (ESM) [16], or only rely on cytosine methylation signals, such as MethylSeekR [24], Methpipe [25], and MMSeekR [26]. As a result, the biological state annotations differ between these two classes of segmentation tools and are rarely combined. For instance, in the context of chromatin segmentation, terms such as promoter, enhancer, or transcribed regions partially overlap and correspond to DNA methylation states that are categorized as unmethylated, lowly methylated, or highly methylated domains, respectively. Moreover, CpG positions that contain the DNA methylation information are not equally distributed across the genome, generating regions with low or no information content. The generation of an integrative model that effectively incorporates both classes of epigenetic signals and models their interactions would thus offer a more comprehensive and interdependent view on the epigenomic landscape.

As a basis for such an integrated segmentation tool, we use the basic framework of ESM [16], a recently developed segmentation tool that incorporates new features to address some of the limitations of existing tools. First, ESM has a broader flexibility to model the distribution of epigenetic marks by not only supporting the conventional Poisson or Negative Binomial distribution but also a wide range of other discrete probability distributions with varying flexibility to model variance and skewness of the histone count data. As a consequence, it allows us to better capture the distinct distributional properties of histone mark counts, such as the often observed overdispersion of H3K27me3 or H3K4me1 counts. Second, built-in flexible duration modeling efficiently captures short-duration (nucleosomal) and long-duration (domain) states simultaneously in a single model.

We here present an extended version of ESM, which we name EpiSegMixMeth (ESMM). ESMM is a comprehensive epigenomic segmentation tool addressing the challenges for an integrated probabilistic model for a truly combined and integrated use of genome-wide chromatin data and DNA methylation data. We apply ESMM to a large and uniquely processed full epigenome dataset provided by the IHEC Human Epigenome Atlas (unpublished data). To our knowledge, ESMM is the first tool to support such a read count-based simultaneous integration of different epigenetic modalities. We show that ESMM enhances the cell-type-specific classification of epigenomes, particularly in large, previously not well captured and annotated heterochromatic regions. As an example, we show how ESMM data can be used to follow epigenomic state transition in developing cells, revealing new interpretation links to three-dimensional Hi-C data.

## Materials and methods

### General framework of ESMM

Our latest work introduced ESM as a flexible hidden Markov model to discover chromatin states from histone mark data [16]. Since histone mark information is typically obtained from ChIP-seq experiments that, after alignment, generate overdispersed and skewed count data, ESM allows the read counts of each histone modification to follow a different discrete distribution type, supporting a wide range of discrete distributions, such as the Poisson, Binomial, Negative Binomial, Beta Negative Binomial, and their corresponding zero-inflated versions.

The extended-state topology of the hidden Markov model used by ESM further allows us to model state durations that follow a Negative Binomial distribution instead of a Geometric distribution. This is particularly important for modeling long heterochromatic domains and allows us to capture simultaneously short- and long-duration states.

In this work, we extend the framework of ESM to also handle DNA methylation data, e.g., from whole genome bisulfite sequencing. Due to its flexible distribution modeling, it is a natural choice to extend the distribution types supported by ESM to handle the specific properties of DNA methylation data.

DNA methylation at a genomic region is typically defined by two counts: the read coverage and the number of methylated reads.

To consider both coverage and methylation information, ESMM fits DNA methylation count data using either the Binomial or Beta Binomial distribution. The probability mass function of a Binomial distribution with success probability $p \in [0, 1]$ and number of trials $n \in \mathbb {N}$ is given by


\begin{align*}
P[X = k] = \binom{n}{k} p^k (1-p)^{n-k} \,\,\,\,\,\,\,\,\ (k=0, 1, 2, \dots , n)
\end{align*}

and the probability mass function of a Beta Binomial distribution with parameters $\alpha > 0, \beta > 0$ and number of trials $n \in \mathbb {N}$ is given by


\begin{align*}
P[X = k] = \binom{n}{k} \frac{B(k + \alpha , n - k + \beta )}{B(\alpha , \beta )} \,\,\,\,\,\,\,\,\ (k=0, 1, 2, \dots , n)
\end{align*}

where $B(\cdot , \cdot )$ is the beta function. To compute the emission probabilities in the hidden Markov model, the value of the random variable $k$ is given by the number of methylated CpGs and the parameter $n$ by the methylation coverage. Thus, only $p$ or $\alpha$ and $\beta$ are estimated for the Binomial and Beta Binomial distribution, respectively. This contrasts with the fitting of histone counts using these distributions, where both $n$ and $p$ or $n$ and $\alpha , \beta$ are treated as parameters that have to be estimated. The fitting of the histone counts and the duration modeling in ESMM is performed exactly as in ESM [16].

### Input data and processing

#### Chromatin immunoprecipitation followed by sequencing, whole genome bisulfite sequencing, and RNA sequencing data preprocessing

We downloaded all epigenomic data from the IHEC repository https://ihec-epigenomes.org/epiatlas/data/, specifically focusing on entries in the Epigenome Reference Registry (EpiRR) marked as complete epigenomes. These datasets include the six key histone marks [chromatin immunoprecipitation followed by sequencing (ChIP-seq) data]: H3K4me3, H3K27ac, H3K4me1, H3K36me3, H3K27me3, and H3K9me3, along with DNA methylation data [whole genome bisulfite sequencing (WGBS)] and gene expression signals [RNA sequencing (RNA-Seq)]. We selected only primary cells designated as healthy, resulting in 154 EpiRR entries that met these criteria. We acquired ChIP-seq BAM and bigwig files, WGBS CpG.bed and bigwig files, and RNA-Seq gene.results and bigwig files from these entries. All data was aligned to the reference human genome (hg38). Using the ESM package [16], we generated the ChIP-seq count matrices by applying a sliding nonoverlapping window of 200 bp to the BAM files. For the WGBS data, we used the BSmooth method [27] to smooth methylated reads per CpG site using a 200 bp sliding window. We then calculated the averages of methylated and unmethylated reads across CpGs for each bin to obtain bin counts (assigning 0 methylation values in the matrix if the 200 bp bin has no CpG) for a total of 15 441 313 bins in the ChIP-seq count matrix. We included counts of chromatin marks and the average methylated and unmethylated read counts for each bin in the final count matrix. For ChromHMM, we binarized the input signals using the BinarizeBam command from the ChromHMM package [28], with the 200 bp window size parameter and hg38 as the reference human genome.

#### Hi-C data preprocessing, normalization, and interaction calling

Hi-C data was obtained from the European Genome-Phenome Archive [29] (accession number EGAS00001004763). The sequencing reads of Hi-C experiments were processed with the nf-core/hic pipeline, version 2.0.0 [30], *genome* set to ’GRCh38’, *digestion* to ’mboi’, *resolution* and *binsize* to 1000, 2000, 5000, 10000, 20000, 25000, 30000, 35000, 40000, 45000, 50000, respectively. Read quality control was performed by FastQC [31]. The main HiC-Pro module of the pipeline performed the mapping (using bowtie2 with a two-step strategy to rescue reads spanning the ligation sites), detection of valid interaction products, duplicate removal, and generation of raw and normalized contact maps. Genome-wide contact maps were created at different resolutions using cooler v0.10.2 [32]. The cooler balancing algorithm was applied to normalize the contact maps [32]. Biological replicates (rep1, rep2, and rep3) were merged with the cooler merge option and default parameters.

#### Compartment and TAD calling

The data was binned using *genome binnify* at the aforementioned resolutions from cooltools v0.7.1 [33]. Afterward, the matrix was corrected for GC bias and very low/high contact regions, with default parameters for *genome gc* from cooltools. Compartments were called with cooltools *call-compartments*. The compartments were called against intra-chromosomal data only instead of genome-wide, with the flag --cis-only; otherwise, default parameters were used. TAD calling was performed with hicFindTADs from the HiCExplorer tool suite v3.7.2 [34–36], with parameters --minDepth 300000 --correctForMultipleTesting fdr.

#### Log2-ratios of normalized interactions

Normalized Hi-C maps of the four B-cell subpopulations were analyzed at different resolutions. Logarithmic ratios of the normalized contact map data were computed between NBC and GCBC, between GCBC and PC and between GCBC and MBC, with the hicCompareMatrices tool from HiCExplorer [34–36].

### Chromatin segmentation and state characterization

#### ESMM, ESM, and ChromHMM segmentation

We used binarized signals of core histone marks from the ChromHMM BinarizeBam command as an input for the LearnModel command from the ChromHMM package to perform ChromHMM segmentation with parameters of binsize 200 bp, numstates 10, and assembly hg38.

As an extended version of ESM, ESMM allows each signal to be fitted independently with a different type of probability distribution in the mixture model. The input for ESM and ESMM is the histone count matrix and DNA methylation level per 200 bp genomic bin (see Input data and processing). We ran ESM and ESMM with duration modeling (online available workflowTopology/Snakefile) using default parameters and setting states to 10. We used the Beta Negative Binomial (BNB) distribution to fit the read counts of all six histone marks. In ESMM, we used the Beta Binomial (BB) distribution to fit DNA methylation data. We selected the Beta Negative Binomial and Beta Binomial distribution due to their high flexibility and good performance in previous experiments.

We trained 154 individual models representing each sample. It is worth noting that we used per-sample training to avoid cohort composition bias that might cause a loss of cell-type-specific signatures in rare or underrepresented cell types. Individual training also enhances robustness against technical inter-sample variability due to variability in data qc and methylation levels across samples. Next, following our previous workflow [16], the number of chromatin states was set to 10 based on the following criteria: (i) manual inspection of biological relevance across a subset of samples, and (ii) model selection using log-likelihood, AIC, and BIC evaluated on subset of B and T cell samples. For the reduced model (ESMM, ESM, and ChromHMM), we performed the same steps, excluding H3K9me3 and H3K27me3 from the input count matrix for each model, and used only B- and T-cell cell epigenomes (40 samples).

#### Annotating segmentation states with relevant functional labels

For each sample, we constructed an enrichment overlap incorporating 23 features derived from three models: ESMM, ESM, and ChromHMM. The analyzed features included mean overlap counts for histone modifications H3K4me3, H3K27ac, H3K4me1, H3K36me3, H3K27me3, and H3K9me3; methylation ratio; genomic coverage of each state; CpG density; mean gene expression; and overlaps with genes, exons, transcription start sites, transcription end sites (TES), transcription start site (TSS) 2 kb upstream, partially methylated domains (PMDs), and various families of repeat elements such as SINE, RNA, Retroposon, LINE, LTR, RC, and Tandem repeats. We normalized these features using a min-max approach for each sample to calculate an enrichment score ranging from 0 to 1. Utilizing these scores, we manually assigned functional labels to each of the 10 states across 29 samples, each representing a different cell type. We employed a random forest classifier [37] in R (4.3.1), training it on these 290 data points with the ‘ntree’ parameter set to 100. To guarantee unique labeling within each sample, we utilized the Linear Sum Assignment Problem’s [38] (LSAP) cost matrix. For states assigned with low probability, we designated exclusive labels for the sample within the models.

### Qualitative and quantitative comparison of segmentation tracks

#### Finding state overlaps and epilogos

To calculate the state overlap between ESMM and ChromHMM’s *no signal* state, we counted each 200 bp bin marked as the ChromHMM *no signal* state across any of the 154 samples. We then overlaid the ChromHMM-NS annotated bedfile with each ESMM state for all samples and calculated the mean overlap counts for each state across all samples. To assess the importance of states in ESMM and ChromHMM using saliency features based on information entropy, we utilized Epilogos [39] with the single mode (–single) and saliency level 1 (S1 - Kullback-Leibler relative entropy). We subsequently visualized the output from Epilogos using pyGenomeTracks [40] around the *AICDA* locus (chr12:8550 000–9650 000).

#### Predicting gene expression using RNA-Seq

To assess the predictive power of gene expression based on segmentation labels from each model, we overlapped the gene.results file for each sample, assigning each bin its log-transformed gene activity (Fragments Per Kilobase of transcript per Million mapped reads - FPKM). Importantly, predictions were generated using the random numbered states (1–10) output by each model, without applying biological labels. This approach ensured that gene expression evaluation was independent of the labeling step. We then split the data into training and testing sets in a 70%–30% ratio, respectively, and trained a linear model to predict gene activity using labels (numbered states) as categorical variables. Finally, we quantified and reported the model’s accuracy using the coefficient of determination (R^2^) and root mean square error of the test set.

#### Complete vs. reduced model analysis

To measure the similarity between states defined by the complete and reduced models, we employed the Jaccard index from bedtools [41] as a similarity score. For each state in the reduced model, we assigned the corresponding state in the complete model that had the highest Jaccard index, ensuring that each label was used only once per sample. Additionally, to generate data for Sankey plots, we counted the combinations of state pairs from the reduced and complete models for all 200 bp bins across the dataset.

#### Consistent annotation within same samples of heterogeneous quality

We analyzed eight samples of luminal epithelial cells, which varied in quality based on the H3K27me3 metric. We categorized these samples into three groups: low, mid, and high quality. As we used samples derived from the same cell type, we treated them as biological replicates and expected them to have consistent segmentation annotations, despite differences in quality. For each EpiRR, we calculated the proportion of all bins marked as facultative heterochromatin and plotted this proportion against the quality of H3K27me3, measured by Jensen–Shannon distance, in increasing order. Furthermore, we fit a smooth linear regression line between the genomic coverage of facultative heterochromatin and the quality metric of H3K27me3, and plot this regression line along with the variance for both the ESMM and ESM models. To assess the consistency of annotation in samples of luminal epithelial cells, we calculated the Jaccard index score for each model (ESMM and ESM) between the same state for each pair of samples. Furthermore, we calculated the difference in similarity scores for each state between the models as JI$_{ESMM}$  $-$ JI$_{ESM}$. A difference $> 0.2$ indicates that ESMM provides consistent annotations, while an absolute difference of $\le 0.2$ suggests that both ESMM and ESM perform comparably well.

#### Inter- and intrastate interactions

We overlapped Hi-C data with our state annotation to understand the interactions of states in a 3D space. For each B-cell type, we used a 200 bp resolution of normalized Hi-C data. We considered a contact valid if a 200 bp bin has a contact to another 200 bp bin in the normalized Hi-C sparse matrix (.cool files), and the linear distance between these bins is not greater than 4 Mb (megabases). We then annotated each valid contact to and from a 200 bp bin using the segmentation annotations generated by both ESMM and ChromHMM.

#### Gene ontology using ESMM-defined state transitions

We overlapped the bins annotated as Het_F in naive B-cells (NBC) as well as germinal center B-cells (GCBC) with Enh_Wk in memory B-cells (MBC). We used the coordinates of these overlapped bins in the EpiRegio database to find the genes associated with or affected by these regions. Using the list of genes, we performed Gene Ontology analysis using gprofiler [42] and filtered only terms related to biological processes. We further sorted them using the combined rank of $log_{10}$ adjusted *p*-value and precision (proportion of genes for the terms).

## Results

### ESMM: An integrative segmentation tool for histone and methylation data

To generate an integrated segmentation model, we combined approaches developed for DNA methylation segmentation with our recently developed chromatin based tool ESM [16]. In short, we constructed a flexible extended hidden Markov state duration model that incorporates both histone modifications and DNA methylation sequencing data on a read count-based level (see the ‘Materials and methods’ section for further explanation). Epigenetic signals were captured in nonoverlapping windows of 200 bp. Histone mark counts were obtained by taking the number of reads mapping to a window, while for DNA methylation, both CpG coverage and methylated CpGs falling into a window were considered.

**Figure 1.**
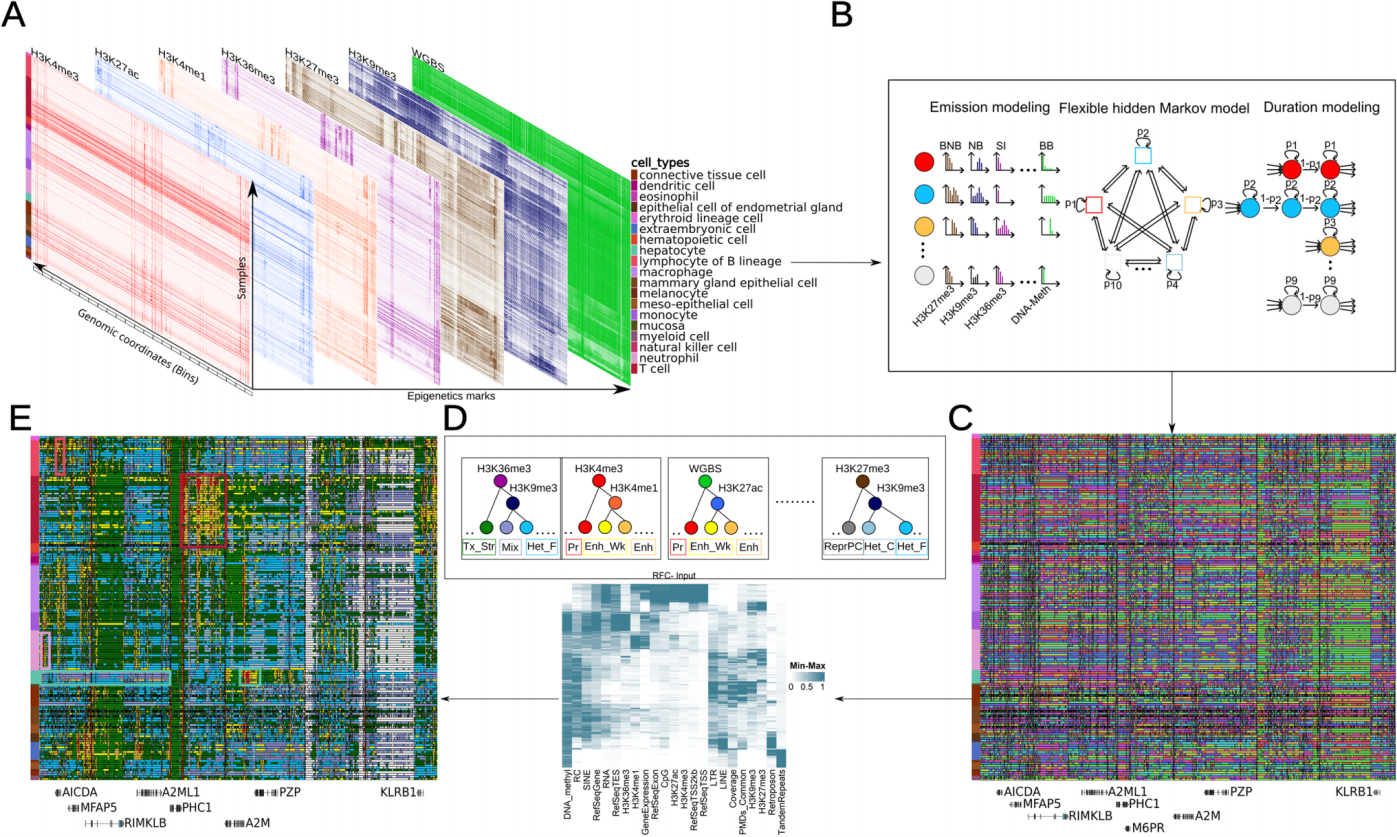
Schematic overview of the joint-model and annotation strategy. (**A**) Histone modifications and DNA methylation signals across 154 human epigenomes are used as input for the segmentation model. Samples span a wide range of primary tissues and cell types. Segmentation was performed using the human hg38 reference genome and a bin size of 200 bp. The region shown corresponds to *AICDA* gene locus (chr12:8550 000–9650 000). (**B**) The ESMM model fits an HMM with mixture and duration modeling, trained independently for each sample. (**C**) Chromatin states across 154 epigenomes in *AICDA* locus using 10-state ESMM model. State labels are assigned arbitrarily per sample. (**D**) A random forest classifier was applied to each sample to map the numerical state labels to unified, biologically meaningful chromatin states. (**E**) *AICDA* locus for the same samples post uniform labeling, providing clear cell-type-specific states as well as agreement between samples of the same cell and tissue type. Pink, red, and light purple boxes surround active regulatory states for B-, T-cells, and macrophages, respectively. Turquoise box encapsulates liver-specific repressive states (left) and liver-specific active regulatory states (right).

To evaluate the performance and robustness of ESMM, we applied it to 154 complete, nonimputed epigenomes from healthy human cells or tissues, uniformly processed and obtained from the IHEC EpiATLAS [4]. Each complete epigenome consists of WGBS, RNA-Seq and six core histone marks: H3K4me3, H3K27ac, H3K4me1 (narrow marks), H3K36me3 (transcription mark), and broad repressive marks H3K27me3 and H3K9me3. The sample set spans multiple tissues and primary cells grouped into 19 distinct cell types (see Fig. 1A), including a large set of immune cells (see Supplementary Fig. S1). While some variability in signal quality and quantity was observed (see Supplementary Figs S2 and S3A–C), the overall data quality was deemed sufficient for integrative modeling.

Each of the 154 samples was modeled independently. Based on empirical testing with different numbers of states, we found that a 10-state model provided the most stable and biologically interpretable segmentation across all samples.

### Generation of unified ESMM state annotations across epigenomes

As a consequence of the individual training for each sample, the ten hidden states are arbitrarily ordered in each trained model. Hence, to enable direct comparison of chromatin states across all 154 samples (see Fig. 1C), we implemented a unified state classification method. We employed a random forest classifier to identify and label the states across all samples (see Fig. 1D and the ‘Materials and methods’ section for more details). This classifier used 23 features, including the average signal and overlap enrichment across six histone marks, DNA methylation, genomic coverage, gene expression, genes, TES, common PMDs [43], and repeat elements [44].

The unified labeling, followed by grouping cell types, revealed distinct cell-type-specific state emission patterns (Fig. 1D). For example, Fig. 1E highlights a genomic region containing genes active in lymphoid cells where we observed strong enrichment of regulatory states (yellow and orange states in the pink, red, and light purple boxes). These include B-cell-specific expression of *AICDA* gene (see Supplementary Fig. S4A), T-cell-specific expression of *KLRB1* [45], and macrophage-specific activity of *CLEC4E* [46], each associated with regulatory chromatin states.

Importantly, these cell-type-specific patterns extended beyond activation signals. We also observed widespread cell-type-specific repressive states (light blue and purple states in the turquoise box), often characterized by an absence of gene expression. Notable examples include clear repressive chromatin configurations in liver cells at immune-cell-specific loci such as *CLEC4E, AICDA*, and others (see Supplementary Fig. S4B).

In summary, the flexible segmentation provided by ESMM enabled the construction of a comprehensive, high-resolution map of chromatin states across diverse cell types. It successfully captured both activating and repressive patterns, offering key insights into cell-type-specific epigenomic programs.

### Integrative epigenome segmentation recovers large domains missed by histone-only models

**Figure 2.**
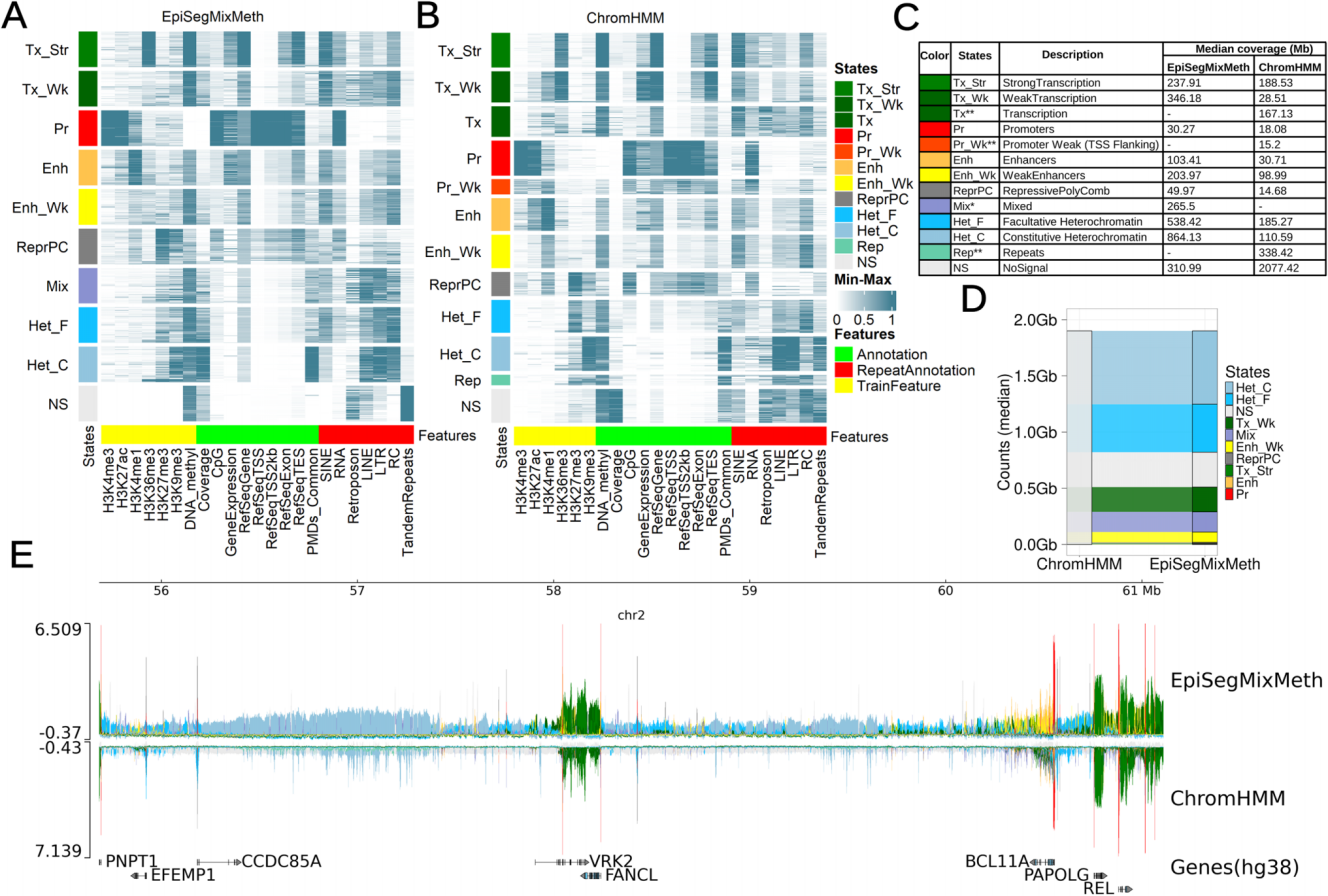
Epigenomic-wide state characterization of 154 samples. (**A**) ESMM states defined using six core histone marks and DNA methylation annotated with biological functions. Labels are assigned based on the random forest classifier. Features on the x-axis are grouped into *train* (yellow), which are used in the model; *annotation* (green); and *repeat annotation* (red). (**B**) ChromHMM states from models trained only on the six core histone marks, labels were assigned as in panel (A). Note that a large part of the genome is assigned to the No Signal state (NS). (**C**) States with their color code, biologically associated function, and median genomic coverage for both ESMM and ChromHMM in Megabase (Mb). ChromHMM-specific states are denoted with **, while ESMM-specific states are denoted with *. (**D**) Overlap between ESMM states and ChromHMM NS state, showing reclassification of ChromHMM-NS states to various functional biological states in ESMM. (**E**) Epilogos: a multibiosample functional genomic annotations view around a large heterochromatic domain locus (chr2:43 832 201–62 396 766) for ESMM and ChromHMM. *Y*-axis shows the S1 score of epilogos for ESMM whereas the inverted *y*-axis for ChromHMM. S1 quantifies deviation of chromatin state frequency from the genome-wide average; higher scores indicate atypical/important patterns across biosamples.

A comparative analysis of the genome-wide state enrichment across 154 samples reveals that ESMM performs well not only in gene-rich regions and regulatory spaces but also in large parts of the genome that ChromHMM, a histone-only-based segmentation model, assigns the *quiescent* or *no signal* state (see Fig. 2A and B). ESMM segments a large proportion of these previously unannotated regions to various functional heterochromatic states (see Supplementary Fig. S4B as an example region). We notice that ChromHMM defines exclusive states that exhibit a lower certainty for all labels during classification. We annotate these three ChromHMM-exclusive states (marked with an asterisk in Fig. 2C) as transcribed (Tx), weak promoter (Pr_Wk), and repeat (Rep) states. In comparison, ESM (see Supplementary Fig. S5) and ESMM define a specific (mixed) state not covered by ChromHMM. In total, the comparison between ESMM and ChromHMM comprises 13 distinct functional states. The exclusive ChromHMM states Tx, Pr_Wk and, Rep are submerged in other states of ESMM leading to clusters of regulatory domains with slightly variable sample counts but highly similar patterns. Despite this seemingly lower granularity, ESMM is best at differentiating between low and high transcriptionally active genes (see below). The high transcribed state is marked by high H3K36me3 and high DNA methylation levels. This Tx_Str state covers relatively larger domains (see Fig. 2A). ESMM utilizes overlapping features of chromatin and DNA methylation and detects a significant co-occurrence of features and terminologies provided by chromatin and DNA methylation [e.g. unmethylated regions (UMRs) and lowly methylated regions (LMRs)] based segmentations. We noticed that the Tx state defined by ChromHMM is inconsistent between samples, which is reflected in the genomic coverage in Fig. 2B and the Tx_Wk state shows relatively low occupancy.

The promoter state (Pr) is consistently captured in all models, in line with a very low methylation in CpG dense promoter regions (UMRs) and the highest enrichment of the signature promoter marks H3K4me3 and H3K27ac compared to all other states. Both, ESMM and ChromHMM, define two enhancer states (Enh and Enh_Wk) with apparent differences in H3K4me1 and DNA methylation levels. Weak enhancers show high DNA methylation levels and a reduced/depleted H3K4me1 enrichment. Overall, we observe a very good agreement between ChromHMM and ESMM euchromatin-specific states, including transcription and regulatory states, which cover a relatively small part of the genome together.

A major difference between ESMM and ChromHMM emerges as ESMM provides a much broader coverage and clearer distinction of heterochromatic states. The repressive polycomb (ReprPC) state is marked by high H3K27me3 and an intermediate level of DNA methylation and CpG density (see Fig. 2A). ESMM features a novel mix state comprising a combination of the repressive marks H3K27me3 and H3K9me3, an enrichment of repeat elements, and a moderate DNA methylation. The facultative heterochromatin state shows a relatively lower enrichment for H3K27me3, reduced DNA methylation including some common (shared) PMDs, and favors rolling circle repeats (RC). While ChromHMM also identifies these states, the enrichment is less pronounced, and the genomic distribution and coverage appear more unstable. The most repressed state, constitutive heterochromatin (Het_C), shows the highest enrichment of H3K9me3, a constant high CpG-methylation signal, and an enrichment of long terminal repeats (LTRs). Constitutive heterochromatin shows an almost exclusive enrichment of common (shared) PMDs (defined in [43]) pointing towards a substantial overlap between ESMM and MethylSeekR, a methylation-based segmentation approach.

In summary, ESMM enhances the quality and quantity of state assignments throughout the genome. The proportion of the genome classified as *no signal* state is reduced from up to 70% by ChromHMM to about 10% (see Fig. 2D and, as an example, Supplementary Fig. S8). ESMM not only enhances the overall calling of heterochromatic segments (Fig. 2E), it also differentiates no-signal states into either constitutive or facultative heterochromatin or a transitional mix state. Overall, ESMM provides an enhanced and robust genome-wide classification of heterochromatin, not only in large heterochromatic domains but also in smaller intergenic regions, as can be seen in the generally higher Epilogos scores associated with a facultative heterochromatin (and mix) state (see an example in Fig. 2E).

### DNA methylation supports robust calling of eu- and heterochromatic states

**Figure 3.**
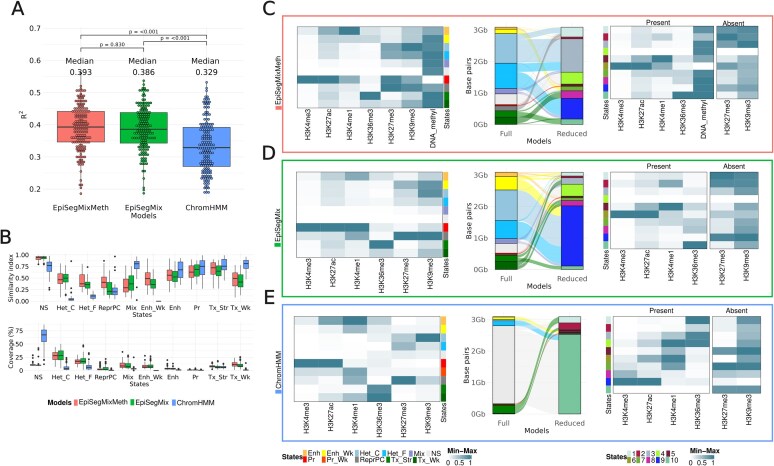
Contribution of DNA methylation to the mixture model segmentation. (**A**) Dotplot and boxplot measuring predicted gene expression (R-square score) accuracy using functional states defined by three different models. (**B**) The upper panel shows genome-wide similarity. The Jaccard index measured between the same states, defined using the full and reduced models for 40 B- and T- cell samples. The complete model consists of all training features used in each respective model, i.e., six core histone marks (H3K4me3, H3K27ac, H3K4me1, H3K27me3, H3K36me3, and H3K9me3) and DNA methylation for ESMM whereas six core histone marks for ESM and ChromHMM. The reduced model removes the broad repressive marks H3K27me3 and H3K9me3. The similarity index represents the fraction of each state that can be captured efficiently even in the reduced model compared to the full model. The lower panel shows the genomic coverage of the corresponding state in a full model across all samples. (**C**) left: NBC heatmap showing enrichment of the training features for a complete model with 10 functional states defined by ESMM, middle: Sankey plots displaying overlap in base pairs between states defined by the full and reduced models, right: NBC heatmap for reduced model states defined by ESMM with additional enrichment of absent marks in the model marked as Absent. (**D, E**) Same as panel (C) for defined states between full and reduced model using ESM and ChromHMM, respectively.

We conducted several analyses to better understand and assess the contribution of DNA methylation into ESMM. First, we evaluated gene expression prediction accuracy (measured by R-square) based on functional chromatin states inferred from three different models: ChromHMM, ESM, and the integrative ESMM model. As shown in Fig. 3 A, ESMM showed a slight improvement over ESM and a more substantial performance gain compared to ChromHMM. Next, we investigated to what extent DNA methylation substitutes for missing heterochromatic marks. For this, we used a dataset of 40 high quality T- and B-cell immune cell samples and compared model performance under two settings: (i) complete input with all six histone marks, and (ii) het-reduced input missing H3K27me3 and H3K9me3 (the heterochromatin marks). We compared the state assignments from the reduced models of ChromHMM, ESM, and ESMM to those from their respective complete models using the Jaccard similarity index and genome-wide coverage.

Despite the absence of heterochromatin marks, ESMM accurately recovered heterochromatic and no-signal states (see Fig. 3B). It also maintained high fidelity in detecting weak enhancers and transcriptional states, suggesting that DNA methylation provides compensatory information, particularly in regions associated with heterochromatin and euchromatin.

Notably, for most chromatin states, ESMM in the reduced setting exhibited the highest overlap with the full model among all methods tested. Each state in the reduced ESMM model corresponded clearly to a specific state in the complete model–a consistency not observed with ESM or ChromHMM (Fig. 3C–E; shown for a representative NBC sample).

Moreover, the emission matrix of the reduced model of ESMM displays a similar enrichment pattern as the complete model, even for the marks excluded from training (H3K9me3 and H3K27me3; Fig. 3C). This illustrates the potential of DNA methylation to compensate for absent heterochromatic marks and accurately recover key repressive chromatin states, including repressive polycomb (ReprPC), facultative heterochromatin (Het_F), and constitutive heterochromatin (Het_C).

Although ESM without DNA methylation can already recapture the active state to some extent, it failed to differentiate between the different classes of closed chromatin states. Instead, it assigns all of them, along with the weak enhancer state, to one state, as shown in the Sankey plot in Fig. 3D. ChromHMM, in the reduced model, categorizes –depending on the cell type– up to 70% of the genome into one state (*no signal*), overlapping with the *no-signal* states, facultative and constitutive heterochromatin states in the full model.

Having shown that DNA methylation can be a proxy for the absence of other chromatin marks, we next examined whether DNA methylation improves segmentation stability, e.g., by compensating for an inconsistent or lower quality of ChIP-seq signals (Supplementary Fig. S2). To demonstrate this, we analyzed segmentation results in relation to the variation in the quality of H3K27me3. We compared the individual sample state performance against robustness score using the Jaccard similarity index and genomic coverage for states affected by this mark in a set of luminal epithelial cells from mammary gland samples.

We observe that the states defined by ESMM are more stable and show less variability against the quality changes of H3K27me3 as compared to a segmentation by ESM or ChromHMM Supplementary Fig. S6A). The genomic coverage of ESM, defining facultative heterochromatin, ranges from 1% to 50% for the low-quality H3K27me3 samples [below 30 Jensen–Shannon Distance (JSD) score], while ESMM produces a more stable annotation of these states ($7\%-30\%$). We observed that the overall variance for the proportion of Het_F state varied primarily based on the JSD score and was more prevalent in ESM than in ESMM. As an example, we highlight (see Supplementary Fig. S6A) a set of luminal epithelial cells of the mammary gland. In our initial QC we noticed that these samples fall into sets of low, mid, and high quality levels across eight replicates. Interestingly, the proportions of the heterochromatin state were robust across all replicates for the ESMM mixture model, while ESM only captured them accurately and agreed with ESMM for high-quality samples. We also noticed that across all replicates of these luminal epithelial cell samples, the replicability for nine out of ten states was higher for ESMM (see Supplementary Fig. S6B).

Overall, we conclude that the integration of DNA methylation in ESMM allows us to robustly capture the cross-talk between different layers of epigenetic marks and enhances the definition of modeled states in previously nondefined regions.

### ESMM uncovers cell-type-specific regulatory transitions during B-cell differentiation

**Figure 4.**
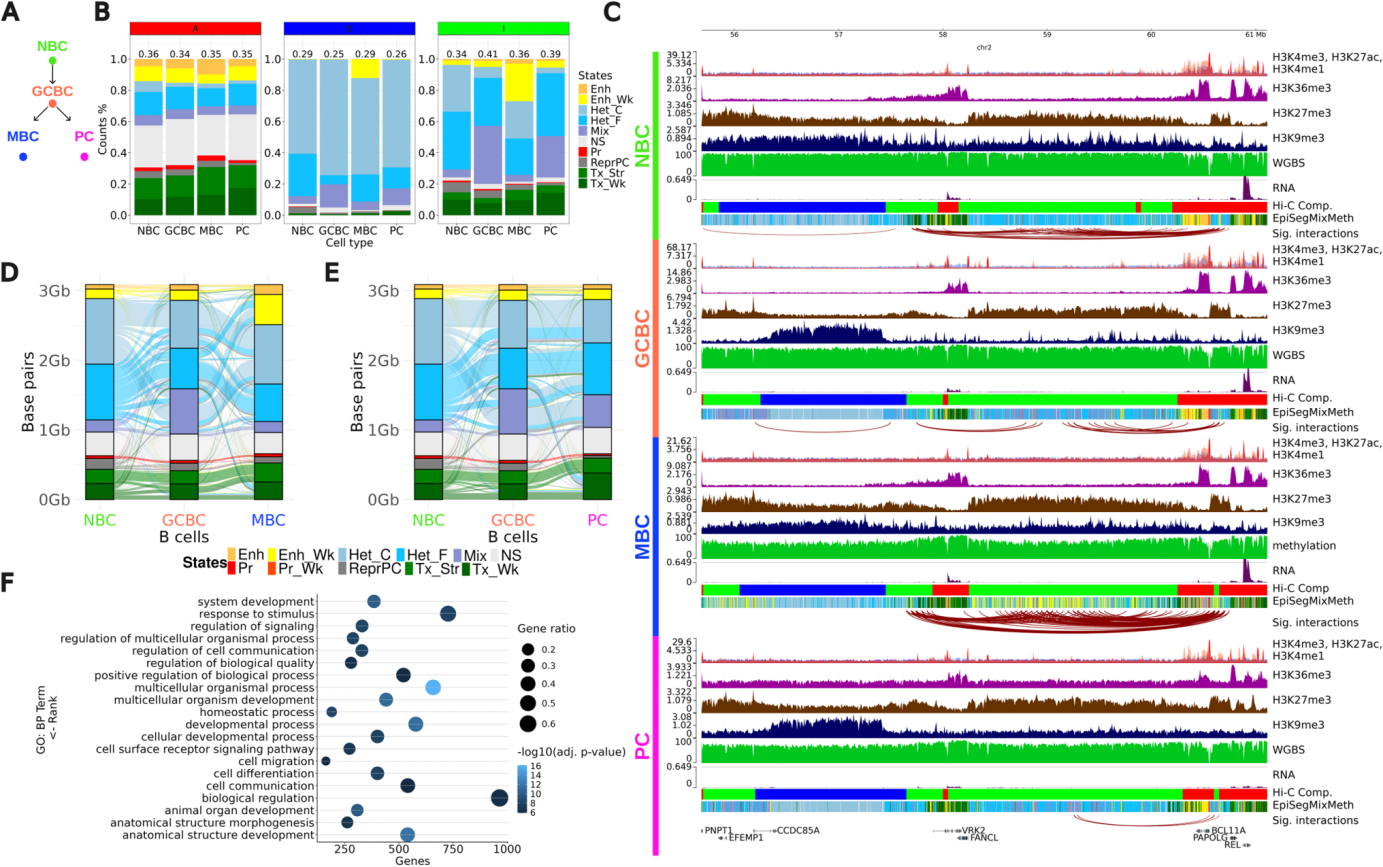
Analysis of state transitions during B-cell differentiation. (**A**) Current model of B-cell differentiation. (**B**) Distribution and proportional changes of ESMM states within 3D compartments (Active ‘A’: red, Inactive ‘B’: blue, Intermediate ‘I’: green) during differentiation. (**C**) Maps of segmental and epigenomic changes during B-cell differentiation across B-cells in the *VRK2/Rel* region: Ordering from top to bottom for each cell type: core histone modifications (narrow marks H3K4me3, H3K27ac, and H3K4me1 overlayed into a single track), WGBS methylation data (0%–100%), RNA-Seq expression levels (log1p normalized), 3D chromatin state segmentation, ESMM segmentation. The arcs below show local significant (max 1 MB distance) interactions based on Hi-C contact map (thickness signifying importance). (**D**) Sankey diagram depicting the state transitions in ESMM from NBC to GCBC to MBC, with the *y*-axis showing genomic coverage and proportional transitions between states. (**E**) The state transition diagram from NBC through GCBC to PC, similar to panel (D). (**F**) Gene Ontology analysis for genes affected by facultative heterochromatin to weak enhancer transition in MBC, selected based on EpiRegio database (see the ‘Materials and methods’ section).

The role of complex epigenetic changes during the B-cell differentiation process has been a matter of intensive studies [20, 47]. Based on various data (including Hi-C based 3D data) a model was proposed (Fig. 4A) that summarizes the stepwise epigenomic transition from NBC to GCBC branching into MBC, and plasma cells (PC) [20]. We speculated that by combining our ESMM based segmentation with matching Hi-C based segmentation data, we should get deeper insights into global and local epigenomic changes accompanying B-cell maturation.

To assess how ESMM-based segmentation aligns with 3D genome architecture, we compared it to Hi-C derived A, B, and I compartments in the same B-cell differentiation states (Fig. 4B). The A and B compartments displayed relatively consistent genome coverage across cell types, ranging from 34%–36% for A and 25%–29% for B, whereas the I compartment showed broader variability (34%–41%), reflecting its dynamic nature during B-cell maturation. Consistent with earlier findings [20, 47], the I compartment emerged as the most variable and responsive to differentiation-related changes. Notably, this compartment was enriched in ESMM-defined chromatin states associated with regulatory activity and heterochromatin remodeling: weak enhancers (Enh_Wk), constitutive heterochromatin (Het_C), and facultative hetero-chromatin (Het_F) in MBC; and mixed states (Mix) along with Het_F in GCBC and PC. These patterns indicate that the I compartment serves as a hotspot for chromatin remodeling, with ESMM providing a finer-grained and functionally informative view complementing the coarse Hi-C compartmentalization view.

A deeper examination of specific genomic regions further highlights the added resolution of ESMM. In MBC, ESMM identified numerous small segments classified as weak enhancers (Enh_Wk) within the ‘I’ compartment. One such region, encompassing the Rel (NF-$\kappa \beta$) and *VRK2* genes-key regulators in MBC function-exhibited a transition from facultative heterochromatin (Het_F) in NBC to a mixture of different states in GCBC to dispersed weak enhancers in MBC, back to a mixture of different states in PC. These finer-grained ESMM segments corresponded with increased and localized MBC-specific 3D contacts (Fig. 4 and Supplementary Fig. S10), suggesting the formation of novel regulatory interactions and loops (depicted by arcs in Fig. 4C), linking enhancers to nearby regulatory elements. In addition to enhancer resolution, ESMM revealed broader domain-level chromatin changes. For instance, an expansion of the A compartment near the *VRK2* locus was observed in NBC and MBC but not in GCBC or PC. This shift was accompanied by changes in adjacent heterochromatic regions, including an increase in H3K9me3 signal and a transition from Het_F to Het_C in GCBC and PC. The segmentation patterns suggest lineage-specific regulatory architecture: MBC retains greater similarity to NBC, while PC more closely resembles GCBC in this region. Globally, MBC showed an increased abundance of weak enhancer states compared to NBC, GCBC, and PC (Fig. 4B), and genes linked to these enhancers were enriched in functional categories related to development, signaling, response to stimuli, and cell differentiation (Fig. 4F).

Finally, a direct comparison of segmental changes from GCBC to MBC versus GCBC to PC (Fig. 4D and E) highlights divergent regulatory trajectories. The GCBC-to-MBC transition is marked by an increase in enhancer-like segments and a reduction in the Mix state, consistent with regulatory activation. In contrast, the GCBC-to-PC transition shows an expansion of Het_F, with no notable gain in enhancer activity, suggesting a shift toward chromatin compaction and transcriptional silencing in PCs. Such segmental changes, not clearly captured by ChromHMM, appear as local changes in the 3D contact maps (see Supplementary Fig. S10) and are also observed on the genome-wide level between B-cell types as clear cell-type-specific inter- and intra-segmental contact patterns (Supplementary Fig. S8).

Overall, our analysis shows that ESMM segmentation has the potential to capture a broad spectrum of active and repressive states, both in the long range chromatin context as well as in a local gene-specific context, and allows us to more precisely link epigenomic segmentation to the three-dimensional domain and interdomain interactions in human cells.

## Discussion

### Implications and advantages of combined modeling of epigenomic marks for chromatin segmentation

In this work, we summarize the results of the first integrated and comprehensive SAGA analysis across a diverse set of 154 IHEC-grade epigenomes, utilizing both histone modification and DNA methylation signals. While existing segmentation tools such as ChromHMM, Segway, and EpiCSeg have advanced the analysis of histone modifications, they typically neglect or decouple DNA methylation, limiting the biological insight that can be gained from truly integrated analyses.

To address this, we developed ESMM, an extension of our earlier method ESM [16], which combines a flexible probabilistic modeling framework with state duration modeling and distributional versatility. ESMM incorporates both ChIP-seq–derived histone modification data and WGBS–derived methylation data, without resorting to binarization thresholds that discard quantitative variation. This is a key advantage, especially in low-methylated regions (LMRs) and heterochromatic domains, where DNA methylation carries important cell-type-specific regulatory information, as shown previously [43].

Previous efforts at integrative segmentation have either relied on indirect post hoc combination of methylation and histone data or, in rare cases, simplistic binarization strategies that obscure biologically meaningful variability. For instance, one prior study in mice [48] applied a 50% methylation cutoff per 200 bp window. Such approaches are particularly problematic in regions of PMDs or facultative heterochromatin, where methylation levels vary continuously and have fuzzy patterns. In addition, binarization impairs the comparison of segmentation results across (many) cells, as overall methylation levels vary significantly between certain cell types and disease states [5]. ESMM overcomes these limitations by modeling raw methylation counts directly, enabling quantitative integration of both signal types. Combining the resulting segmentation of ESMM with a random forest classifier enabled us to perform the first comprehensive comparative analysis of 154 primary human cell and tissue samples, which are archived as high-quality complete epigenomes of healthy cells in the IHEC EpiATLAS [4]. This comparative segmentation represents a novel and unique resource to visualize and track cell- and tissue-specific epigenomic features.

### DNA methylation enhances resolution and stability of epigenome segmentation

Conventional histone modification-based segmentation tools classify a substantial portion of the genome (up to $\sim$ 70%) as ‘no signal/quiescent’ state [49,50] leaving a large portion of the genome poorly characterized and unexplored. Among the regions previously labeled as quiescent, ESMM reassigns 60% to heterochromatin, 30% to euchromatin, and only 10% remain as true no-signal regions, demonstrating an enhanced resolution across genomic domains. For example, we observed that integrating DNA methylation not only facilitated a more accurate capturing of constitutive and facultative heterochromatin but also provided a deeper understanding of their cell-type-specific attributes (Supplementary Fig. S9). For many cell- or tissue-specific genes, we find interesting combinations of cell-specific DNA methylation signatures (PMDs and LMRs) and heterochromatic or euchromatic marks. Regions with constitutive heterochromatin, such as those controlling diverse types of allele-specific epigenetic control in all cells, including X-chromosome inactivation, allelic exclusion, and genomic imprinting [51], do not exhibit this variability across cell types and form stable and uniform PMDs.

Importantly, ESMM maintains strong concordance with histone-only segmentation like ESM and ChromHMM, but demonstrates increased stability when ChIP-seq data are noisy or incomplete (see Supplementary Fig. S7, Jaccard index, states%, and meth%). This is particularly true for broad marks such as H3K27me3 and H3K9me3, where ESMM segmentation remains reliable even when these two marks are absent, in contrast to histone-only segmentation tools. In such cases, DNA methylation was sufficient to recapitulate key heterochromatin states with high reproducibility, reflecting the strong correlation between DNA methylation and repressive histone modifications [52–54]. Moreover, ESMM properly captures the small regulatory elements like promoters and enhancers (Supplementary Fig. S7, Jaccard index, states%, and meth%).

Collectively, these findings highlight ESMM as a powerful and scalable tool for comprehensive chromatin state annotation, especially in datasets where histone marks are missing or limited but WGBS data are available. Implemented as Snakemake workflow, ESMM ensures reproducibility and is well suited for large-scale epigenomic data.

### ESMM captures global and local regulatory epigenomic transitions during B-cell differentiation

Building on the annotations provided by ESMM, we used this for a focused analysis of B-cell epigenomes. The role of complex epigenetic changes during differentiation of human B-cells has been studied quite intensively before [20, 47]. Together with 3D data a model was proposed (Fig. 4A) which describes the stepwise transition from NBC to GCBC, MBC, and PC. We speculated that leveraging the existing comprehensive dataset, including full epigenomes as well as matching 3D chromatin interaction data might give us a deeper insights in the epigenomic changes accompanying the differentiation. In particular, we aimed to deeper understand the changes of A, B, and I compartments in differentiating B-cells based on Hi-C data.

While regions marked by constitutive heterochromatin, promoters, and transcribed states largely maintain stable states (with slight shifts in borders), notable transitions occur in regions marked by facultative heterochromatin and *mixed* state. These dynamic regions often transitioned from weak heterochromatic states to weak enhancers, and in some cases reverted back to a repressive configuration, highlighting the plasticity of regulatory domains during lineage commitment. Notably, many of these transitions were only detectable through the integration of DNA methylation into the segmentation model. For example, in the *Rel (NF-$\kappa \beta$), Rel-DT/VRK2* locus, we observed a marked emergence of discrete weak enhancer elements in MBCs. While ChromHMM broadly annotated this region as a single weak enhancer domain, ESMM was able to resolve it into multiple smaller elements, each associated with distinct methylation and chromatin features. This finer resolution provided by ESMM better captures the functional modularity of regulatory elements during B-cell maturation. ESMM-identified weak enhancers co-localize with an increased number of MBC-specific chromatin loops, suggesting they are functionally linked to promoter regions of flanking genes. This spatial association further supports the regulatory relevance of these newly resolved enhancer elements.

Hi-C data revealed that regions previously annotated as ‘quiescent’—using histone-only segmentation models—can be more accurately reclassified into biologically relevant states using ESMM. As illustrated in Supplementary Fig. S8, these regions exhibit extensive long-range interactions with various active chromatin states, indicating that biologically meaningful features may have been overlooked under the earlier ‘quiescent’ classification.

To further investigate the relationship between reclassified states from the “quiescent” and 3D genome organization, we analyzed the predictive contribution of ESMM states to lamina-associated domains (LADs) and topologically associating domain (TAD) boundaries. ESMM-defined states overlap substantially with LAD subtypes across 12 cell types [55] (see Supplementary Fig. S11A and B). Constitutive heterochromatin states are enriched for T1 LADs and they are both common features of constitutive repressed domains. This indicates that regions annotated as Het_C are largely embedded within lamina. In contrast, states with weak active (Tx_Wk) or weaker repressive (Het_F) functions overlap with intermediate lamina adjacent T2 LADs. Furthermore, previous studies highlight H3K4me1 (enhancer mark), H3K27ac (active mark), and H3K9me3 (heterochromatin mark) as key histone modifications predicting TADs and Hi-C interactions [56, 57]. Consistent with these results, we found that Enh, Enh_Wk, Het_C, and Het_F are the most predictive features of TAD boundaries (see Supplementary Fig. S12).

Overall, our work demonstrates that ESMM segmentation aligns very well with previous broader segmentations, such as the A/B/I classification from Hi-C data [20], LADs [55], TADs [56, 57], and chromatin-based segmentations. It delivers superior granularity that reveals fine-scale epigenetic transitions often masked in coarser models. This includes gene-specific regulatory contact changes, such as those observed in the SKI gene, a well-known transcriptional regulator in MBC differentiation [58,59]. In summary, our analyses demonstrate that ESMM generates data offering a multiscale view of the genome by defining the most comprehensive genome-wide long-range regional segmentation while also capturing local cell-type-specific regulatory elements and transitions at single-locus resolution, as seen for the differentiating B-cells.

## Conclusion

The use of a flexible read count-based integrated segmentation approach offers an extended high resolution view into cell-specific adaptations on a genome-wide level. By incorporating both histone modification and WGBS DNA methylation data, this method enables the reclassification of up to 70% of the human genome that was previously annotated as quiescent or no-signal by other SAGA approaches. The integration of DNA methylation not only enhances the robustness and stability of chromatin state annotations but also effectively compensates for the absence of key heterochromatic marks. The power of this integrative approach is particularly evident in its ability to resolve fine-scale chromatin changes—such as those observed during B-cell differentiation—when aligned with 3D genome architecture data, revealing dynamic transitions that would otherwise remain obscured.

### Limitations

Our approach relies on epigenomic data of sufficient quality, good coverage, and uniform processing as provided by the EpiATLAS data. Particularly the coverage of DNA methylation data should be sufficient (i.e. $\ge 10$x) to capture the variable distribution of CpG positions across the genome and to quantify local DNA methylation changes with high enough precision. The use of a smoothed window-based approach (200 bp windows) for DNA methylation annotation as applied in ESMM might therefore still lead to an underestimate of local contributions of DNA methylation variation, particularly in regulatory segments.

### Outlook

ESMM is inherently flexible and can be extended to integrate additional count-based epigenomic data. Incorporating data types such as open chromatin assays (e.g. ATAC-seq, NOME-seq, or DNaseI-seq) or direct sequencing approaches that simultaneously capture genomic and DNA methylation information may further enhance model performance. These additional layers of information can provide a more comprehensive view of the epigenomic landscape and enable deeper insights into how cells respond to developmental signals or disease-related changes, particularly in the context of underlying genomic variation. It is worth mentioning that, in preliminary analyses, we successfully tested ESMM on cancer samples and nonhuman datasets such as an ENCODE mouse data (not shown) and observed a very good performance, highlighting the broad versatility of ESMM.

## Supplementary Material

gkag591_Supplemental_File

## Data Availability

EpiSegMixMeth is available as part of EpiSegMix and can be downloaded from https://gitlab.com/rahmannlab/episegmix. EpiSegMixMeth segmentation tracks will be provided as part of the IHEC EpiATLAS data portal: https://ihec-epigenomes.org/epiatlas/data/. Computer code and scripts used for data analysis in this paper is available at https://github.com/epigenetics-sb/EpiSegMixMeth-ESMM (https://doi.org/10.5281/zenodo.20273525).

## References

[1] Cedar H, Bergman Y. Linking DNA methylation and histone modification: patterns and paradigms. Nat Rev Genet. 2009;10:295–304. 10.1038/nrg2540 19308066

[2] Bannister AJ, Kouzarides T. Regulation of chromatin by histone modifications. Cell Res. 2011;21:381–95. 10.1038/cr.2011.22 21321607 PMC3193420

[3] Allis CD, Jenuwein T. The molecular hallmarks of epigenetic control. Nat Rev Genet. 2016;17:487–500. 10.1038/nrg.2016.59 27346641

[4] Bujold D, de Lima Morais DA, Gauthier C et al. The international human epigenome consortium data portal. Cell Syst. 2016;3:496–9. 10.1016/j.cels.2016.10.019 27863956

[5] Stunnenberg HG, Abrignani S, Adams D et al. The International Human Epigenome Consortium: a blueprint for scientific collaboration and discovery. Cell. 2016;167:1145–9. 10.1016/j.cell.2016.11.007 27863232

[6] Du J, Johnson LM, Jacobsen SE et al. DNA methylation pathways and their crosstalk with histone methylation. Nat Rev Mol Cell Biol. 2015;16:519–32. 10.1038/nrm4043 26296162 PMC4672940

[7] Nawaz K, Cziesielski MJ, Mariappan KG et al. Histone modifications and DNA methylation act cooperatively in regulating symbiosis genes in the sea anemone Aiptasia. BMC Biol. 2022;20:265. 10.1186/s12915-022-01469-y 36456984 PMC9717517

[8] Fuks F, Burgers WA, Brehm A et al. DNA methyltransferase Dnmt1 associates with histone deacetylase activity. Nat Genet. 2000;24:88–91. 10.1038/71750 10615135

[9] Atlasi Y, Stunnenberg HG. The interplay of epigenetic marks during stem cell differentiation and development. Nat Rev Genet. 2017;18:643–58. 10.1038/nrg.2017.57 28804139

[10] Brinkman AB, Gu H, Bartels SJ et al. Sequential ChIP-bisulfite sequencing enables direct genome-scale investigation of chromatin and DNA methylation cross-talk. Genome Res. 2012;22:1128–38. 10.1101/gr.133728.111 22466170 PMC3371717

[11] Wu H, Coskun V, Tao J et al. Dnmt3a-dependent nonpromoter DNA methylation facilitates transcription of neurogenic genes. science. 2010;329:444–8. 10.1126/science.1190485 20651149 PMC3539760

[12] Weber M, Hellmann I, Stadler MB et al. Distribution, silencing potential and evolutionary impact of promoter DNA methylation in the human genome. Nat Genet. 2007;39:457–66. 10.1038/ng1990 17334365

[13] Thomson JP, Skene PJ, Selfridge J et al. CpG islands influence chromatin structure via the CpG-binding protein Cfp1. Nature. 2010;464:1082–6. 10.1038/nature08924 20393567 PMC3730110

[14] Okitsu CY, Hsieh CL. DNA methylation dictates histone H3K4 methylation. Mol Cell Biol. 2007;27:2746–57. 10.1128/MCB.02291-06 17242185 PMC1899905

[15] Libbrecht MW, Chan RC, Hoffman MM. Segmentation and genome annotation algorithms for identifying chromatin state and other genomic patterns. PLoS Comput Biol. 2021;17:e1009423. 10.1371/journal.pcbi.1009423 34648491 PMC8516206

[16] Schmitz JE, Aggarwal N, Laufer L et al. EpiSegMix: a flexible distribution hidden Markov model with duration modeling for chromatin state discovery. Bioinformatics. 2024;40:btae178. 10.1093/bioinformatics/btae178 38565260 PMC11026141

[17] Mammana A, Chung HR. Chromatin segmentation based on a probabilistic model for read counts explains a large portion of the epigenome. Genome Biol. 2015;16:1–12. 10.1186/s13059-015-0708-z 26206277 PMC4514447

[18] Ernst J, Kellis M. ChromHMM: automating chromatin-state discovery and characterization. Nat Methods. 2012;9:215–216. 10.1038/nmeth.1906 22373907 PMC3577932

[19] Yaffe E, Tanay A. Probabilistic modeling of Hi-C contact maps eliminates systematic biases to characterize global chromosomal architecture. Nat Genet. 2011;43:1059–65. 10.1038/ng.947 22001755

[20] Vilarrasa-Blasi R, Soler-Vila P, Verdaguer-Dot N et al. Dynamics of genome architecture and chromatin function during human B cell differentiation and neoplastic transformation. Nat Commun. 2021;12:651. 10.1038/s41467-020-20849-y 33510161 PMC7844026

[21] Rao SS, Huntley MH, Durand NC et al. A 3D map of the human genome at kilobase resolution reveals principles of chromatin looping. Cell. 2014;159:1665–80. 10.1016/j.cell.2014.11.021 25497547 PMC5635824

[22] Hoffman MM, Buske OJ, Wang J et al. Unsupervised pattern discovery in human chromatin structure through genomic segmentation. Nat Methods. 2012;9:473–6. 10.1038/nmeth.1937 22426492 PMC3340533

[23] Chan RCW, Libbrecht MW, Roberts EG et al. Segway 2.0: Gaussian mixture models and minibatch training. Bioinformatics. 2018;34:669–71. 10.1093/bioinformatics/btx603 29028889 PMC5860603

[24] Burger L, Gaidatzis D, Schübeler D et al. Identification of active regulatory regions from DNA methylation data. Nucleic Acids Res. 2013;41:e155. 10.1093/nar/gkt599 23828043 PMC3763559

[25] Song Q, Decato B, Hong EE et al. A reference methylome database and analysis pipeline to facilitate integrative and comparative epigenomics. PloS one. 2013;8:e81148. 10.1371/journal.pone.0081148 24324667 PMC3855694

[26] Zheng Y, Ziman B, Ho AS et al. Comprehensive analyses of partially methylated domains and differentially methylated regions in esophageal cancer reveal both cell-type- and cancer-specific epigenetic regulation. Genome Biol. 2023;24:193. 10.1186/s13059-023-03035-3 37620896 PMC10463844

[27] Hansen KD, Langmead B, Irizarry RA. BSmooth: from whole genome bisulfite sequencing reads to differentially methylated regions. Genome Biol. 2012;13:1–10. 10.1186/gb-2012-13-10-r83 PMC3491411 23034175

[28] Ernst J, Kellis M. Chromatin-state discovery and genome annotation with ChromHMM. Nat Protoc. 2017;12:2478–92. 10.1038/nprot.2017.124 29120462 PMC5945550

[29] Freeberg MA, Fromont LA, D’Altri T et al. The European genome-phenome archive in 2021. Nucleic Acids Res. 2022;50:D980–7. 10.1093/nar/gkab1059 34791407 PMC8728218

[30] Servant N, nf-core bot, Ewels P et al. nf-core/hic: v2.0.0-2023-01-12. Zenodo, 2023. 10.5281/zenodo.7556794 (20 May 2025, date last accessed).

[31] Andrews S . FastQC: a quality control tool for high throughput sequence data. Babraham Bioinformatics. 2010, https://www.bioinformatics.babraham.ac.uk/projects/fastqc/ (20 May 2025, date last accessed).

[32] Abdennur N, Mirny LA. Cooler: scalable storage for Hi-C data and other genomically labeled arrays. Bioinformatics. 2020;36:311–6. 10.1093/bioinformatics/btz540 31290943 PMC8205516

[33] Open2C, Abdennur N, Abraham S et al. Cooltools: Enabling high-resolution Hi-C analysis in Python. PLoS Comput Biol. 2024;20:e1012067. 10.1371/journal.pcbi.1012067 38709825 PMC11098495

[34] Wolff J, Bhardwaj V, Nothjunge S et al. Galaxy HiCExplorer: a web server for reproducible Hi-C data analysis, quality control and visualization. Nucleic Acids Res. 2018;46:W11–16. 10.1093/nar/gky504 29901812 PMC6031062

[35] Wolff J, Rabbani L, Gilsbach R et al. Galaxy HiCExplorer 3: a web server for reproducible Hi-C, capture Hi-C and single-cell Hi-C data analysis, quality control and visualization. Nucleic Acids Res. 2020;48:W177–84. 10.1093/nar/gkaa220 32301980 PMC7319437

[36] Ramírez F, Bhardwaj V, Arrigoni L et al. High-resolution TADs reveal DNA sequences underlying genome organization in flies. Nat Commun. 2018;9:189. 10.1038/s41467-017-02525-w 29335486 PMC5768762

[37] Ho TK . Random decision forests. In: Proceedings of 3rd International Conference on Document Analysis and Recognition. Vol. 1. Montreal, Canada: IEEE, 1995, 278–82. 10.1109/ICDAR.1995.598994

[38] Burkard RE, Derigs U, The linear sum assignment problem. In: Assignment and Matching Problems: Solution Methods with FORTRAN-Programs. Berlin, Heidelberg, Germany: Springer. 1980; 1–15.

[39] Quon J, Reynolds A, Tripician N et al. Epilogos: information-theoretic navigation of multi-tissue functional genomic annotations. bioRxiv, 10.1101/2025.06.18.660301, 23 June 2025, preprint: not peer reviewed.

[40] Lopez-Delisle L, Rabbani L, Wolff J et al. pyGenomeTracks: reproducible plots for multivariate genomic datasets. Bioinformatics. 2021;37:422–3. 10.1093/bioinformatics/btaa692 32745185 PMC8058774

[41] Quinlan AR, Hall IM. BEDTools: a flexible suite of utilities for comparing genomic features. Bioinformatics. 2010;26:841–2. 10.1093/bioinformatics/btq033 20110278 PMC2832824

[42] Raudvere U, Kolberg L, Kuzmin I et al. g: Profiler: a web server for functional enrichment analysis and conversions of gene lists (2019 update). Nucleic Acids Res. 2019;47:W191–198. 10.1093/nar/gkz369 31066453 PMC6602461

[43] Salhab A, Nordström K, Gasparoni G et al. A comprehensive analysis of 195 DNA methylomes reveals shared and cell-specific features of partially methylated domains. Genome Biol. 2018;19:1–13. 10.1186/s13059-018-1510-5 30266094 PMC6161375

[44] Smit AFA, Hubley R, Green P. RepeatMasker Open-4.0. 2013–2015. http://www.repeatmasker.org (14 February 2025, date last accessed).

[45] Zhang Z, Bahabayi A, Liu D et al. KLRB1 defines an activated phenotype of CD4+ T cells and shows significant upregulation in patients with primary Sjögren’s syndrome. Int Immunopharmacol. 2024;133:112072. 10.1016/j.intimp.2024.112072 38636371

[46] Wells CA, Salvage-Jones JA, Li X et al. The macrophage-inducible C-type lectin, mincle, is an essential component of the innate immune response to Candida albicans. J Immunol. 2008;180:7404–13. 10.4049/jimmunol.180.11.7404 18490740

[47] Kulis M, Merkel A, Heath S et al. Whole-genome fingerprint of the DNA methylome during human B cell differentiation. Nat Genet. 2015;47:746–56. 10.1038/ng.3291 26053498 PMC5444519

[48] van der Velde A, Fan K, Tsuji J et al. Annotation of chromatin states in 66 complete mouse epigenomes during development. Commun Biol. 2021;4:239. 10.1038/s42003-021-01756-4 33619351 PMC7900196

[49] Gorkin DU, Barozzi I, Zhao Y et al. An atlas of dynamic chromatin landscapes in mouse fetal development. Nature. 2020;583:744–51. 10.1038/s41586-020-2093-3 32728240 PMC7398618

[50] Roadmap EC, Kundaje A, Meuleman W et al. Integrative analysis of 111 reference human epigenomes. Nature. 2015;518:317–30. 10.1038/nature14248 25693563 PMC4530010

[51] Feldman N, Gerson A, Fang J et al. G9a-mediated irreversible epigenetic inactivation of Oct-3/4 during early embryogenesis. Nat Cell Biol. 2006;8:188–94. 10.1038/ncb1353 16415856

[52] Ernst J, Kellis M. Large-scale imputation of epigenomic datasets for systematic annotation of diverse human tissues. Nat Biotechnol. 2015;33:364–76. 10.1038/nbt.3157 25690853 PMC4512306

[53] Murphy AE, Askarova A, Lenhard B et al. Predicting gene expression from histone marks using chromatin deep learning models depends on histone mark function, regulatory distance and cellular states. Nucleic Acids Res. 2025;53:gkae1212. 10.1093/nar/gkae1212 39660643 PMC11879020

[54] Fu K, Bonora G, Pellegrini M. Interactions between core histone marks and DNA methyltransferases predict DNA methylation patterns observed in human cells and tissues. Epigenetics. 2020;15:272–82. 10.1080/15592294.2019.1666649 31509087 PMC7028327

[55] Shah PP, Keough KC, Gjoni K et al. An atlas of lamina-associated chromatin across twelve human cell types reveals an intermediate chromatin subtype. Genome Biol. 2023;24:16. 10.1186/s13059-023-02849-5 36691074 PMC9869549

[56] Sefer E . Hi–C interaction graph analysis reveals the impact of histone modifications in chromatin shape. Appl Netw Sci. 2021;6:54. 10.1007/s41109-021-00396-1

[57] Sefer E . ProbC: joint modeling of epigenome and transcriptome effects in 3D genome. BMC Genomics. 2022;23:287. 10.1186/s12864-022-08498-5 35397520 PMC8994916

[58] Laidlaw BJ, Duan L, Xu Y et al. The transcription factor Hhex cooperates with the corepressor Tle3 to promote memory B cell development. Nat Immunol. 2020;21:1082–93. 10.1038/s41590-020-0713-6 32601467 PMC7442689

[59] Laidlaw BJ, Cyster JG. Transcriptional regulation of memory B cell differentiation. Nat Rev Immunol. 2021;21:209–20. 10.1038/s41577-020-00446-2 33024284 PMC7538181

