# Supplementary material for: Integrated flexible DNA methylation–chromatin segmentation modeling enhances epigenomic state annotation
Source: Nucleic Acids Res. 2026 Jun 16;54(11):gkag591. doi: 10.1093/nar/gkag591 (PMC13270199; doi:10.1093/nar/gkag591)
Supplement: gkag591_Supplemental_File [file gkag591_supplemental_file.pdf]

Supplementary Data

This PDF contains Supplementary Figures S1–S12.

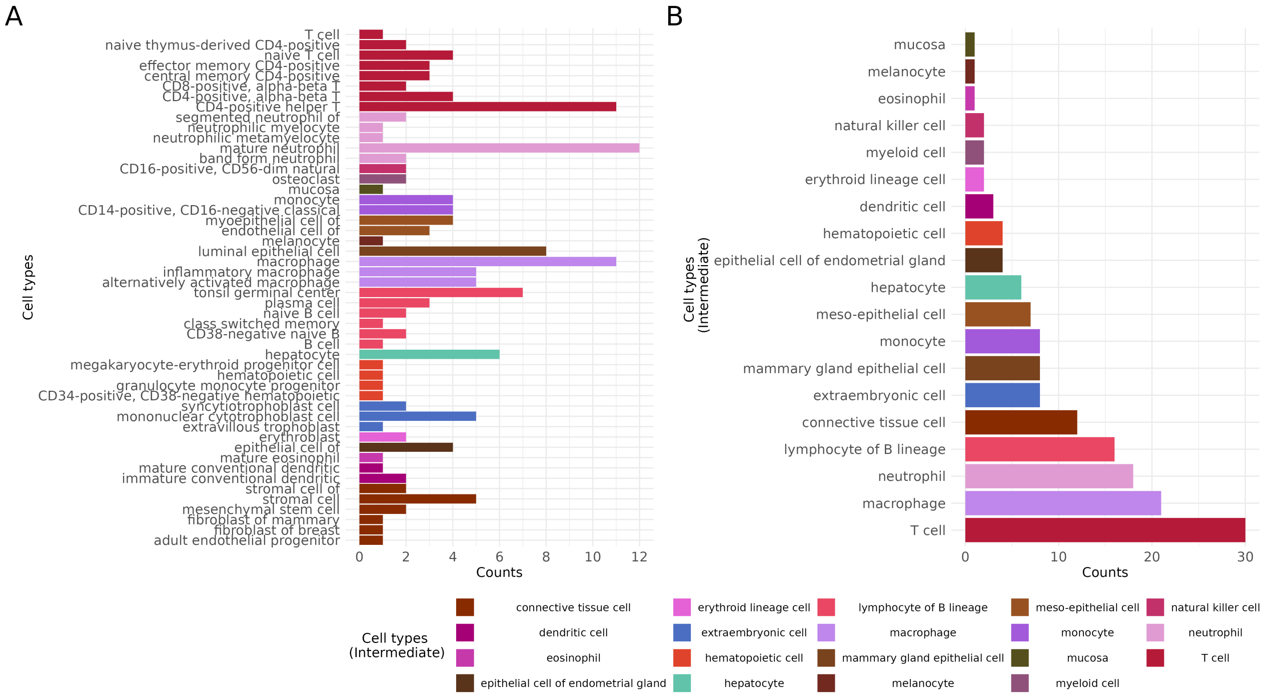

Figure S1 Classification of cell/tissue types across the 154 full epigenome samples of this study. (A) 50 different cell types with lower classification based on IHEC annotation, x axis showing number of samples present for each cell type annotation from various consortia merged in IHEC. (B) IHEC intermediate annotation of the 154 samples into 19 cell/tissue groups used in this study.

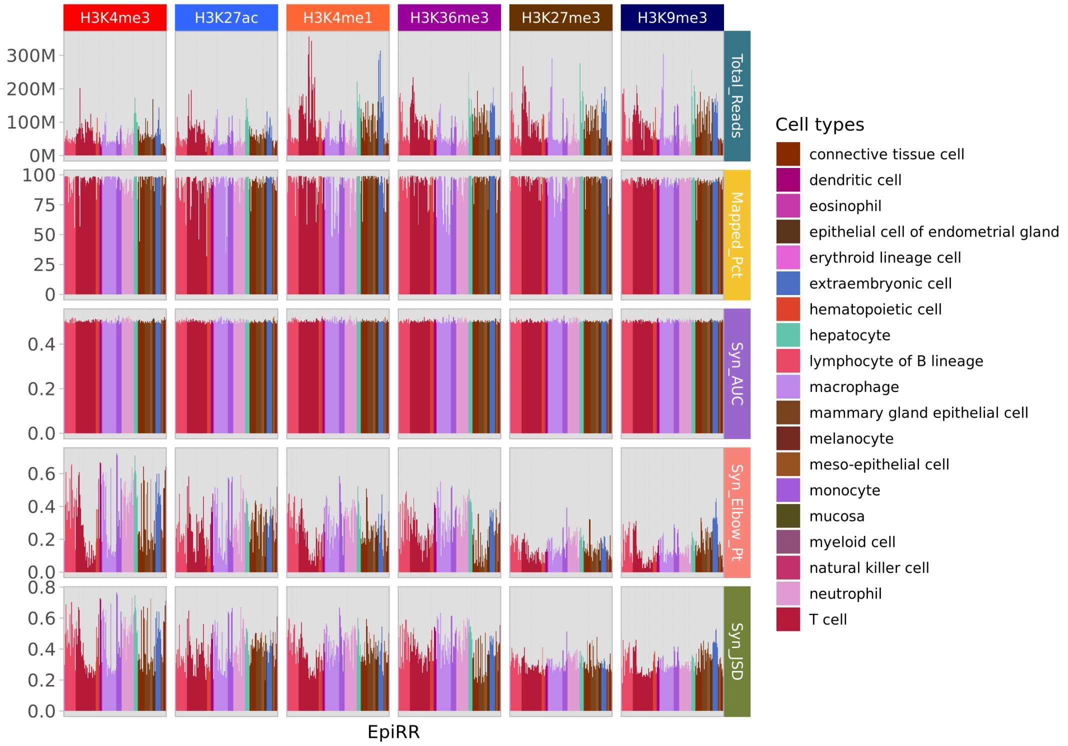

Figure S2 ChIP-seq QC: Five quality control metrics for six core histone marks are shown for 19 cell types used in the study. Top to bottom: total reads in millions, percentage of mapped reads from total reads, synthetic area-under-curve with max value 0.5, synthetic elbow point with a maximum of one and a minimum of zero, and synthetic Jensen-Shannon distance with range $[0, 1]$.

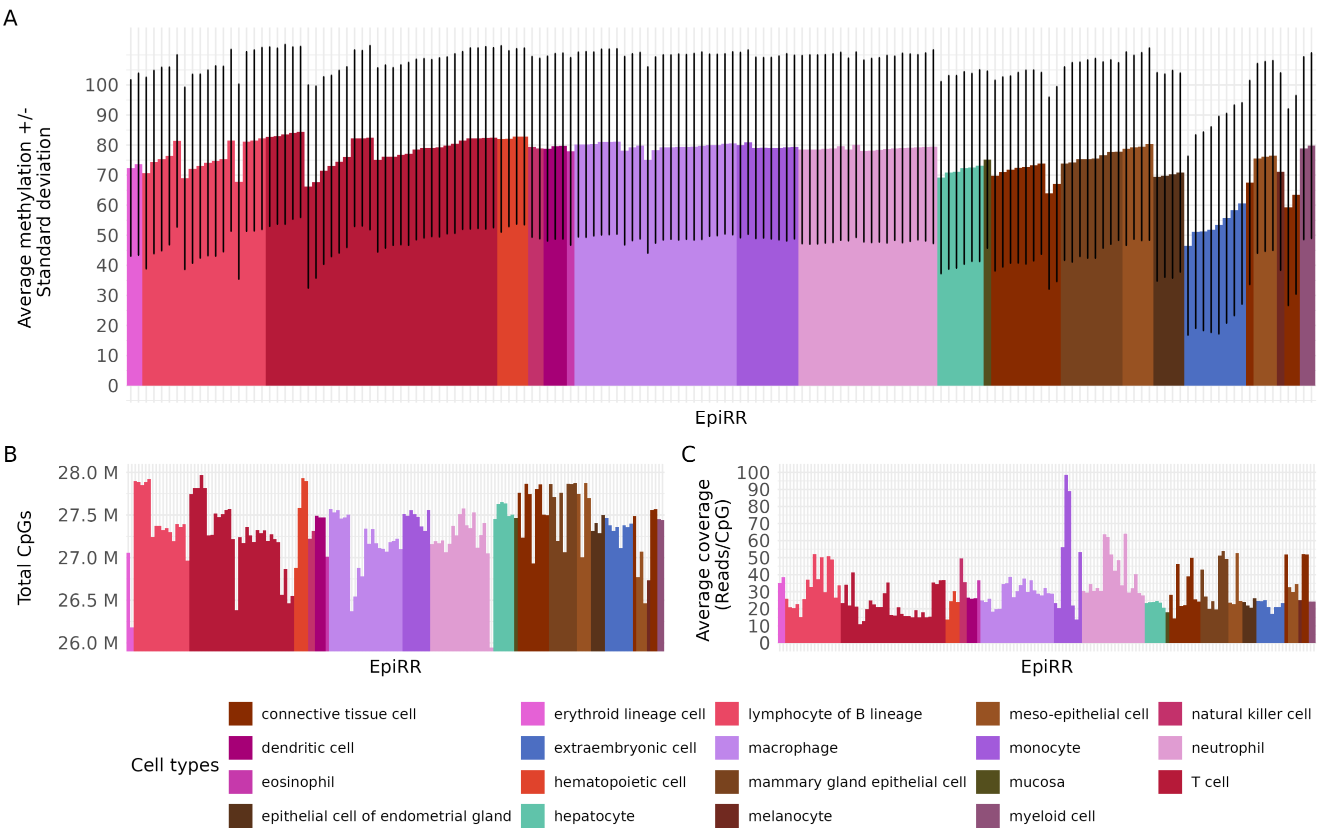

Figure S3 WGBS QC: (A) Average DNA methylation (0–100%) sorted by cell types and increasing average methylation within cell types across 154 samples, capturing cell-type-specific methylation signatures across 19 distinct cell types . (B) Total CpGs in millions covered by WGBS for each sample, categorized by cell type and sorted by increasing average methylation levels, with a minimum of 26 million CpGs in a neutrophil sample. (C) Average sequencing depth/coverage for WGBS data, measuring the average number of reads covered by CpGs with samples in the same order as in (A) and (B), reaching up to 100x coverage for monocyte samples.

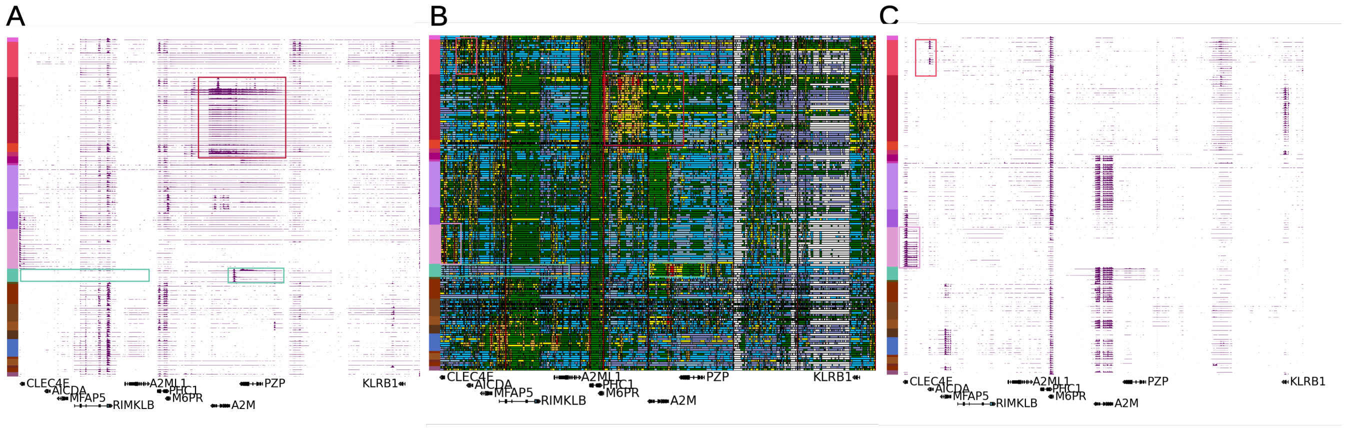

Figure S4 Cell-type-specific segmentation of ESMM in comparison to matching RNA-Seq patterns: (A) Gene expression intensity on the reverse (minus) strand across all 19 cell types (legends for coloring same as S3). The red box demarcates the activity of the A2M gene in a subset of immune cells, and the turquoise box marks cell-specific repression of various genes in hepatocytes. (B) EpiSegMixMeth segmentation tracks in the same order as (A), highlighting the boxed overlay of epigenetic states with RNA-Seq signal. Note the strong expression in (A) accompanied by the accumulation of regulatory states (yellow and red segments). Conversely, the absence of gene activity in hepatocytes (A) with heterochromatin states (blue and purple). The pink box indicates the B-cell-specific activity of the region surrounding the *AICDA* locus, as depicted in (C). (C) Same as (A) but capturing gene expression activity transcribed in the forward strand, with boxes showing cell-type-specific patterns corresponding to (B).

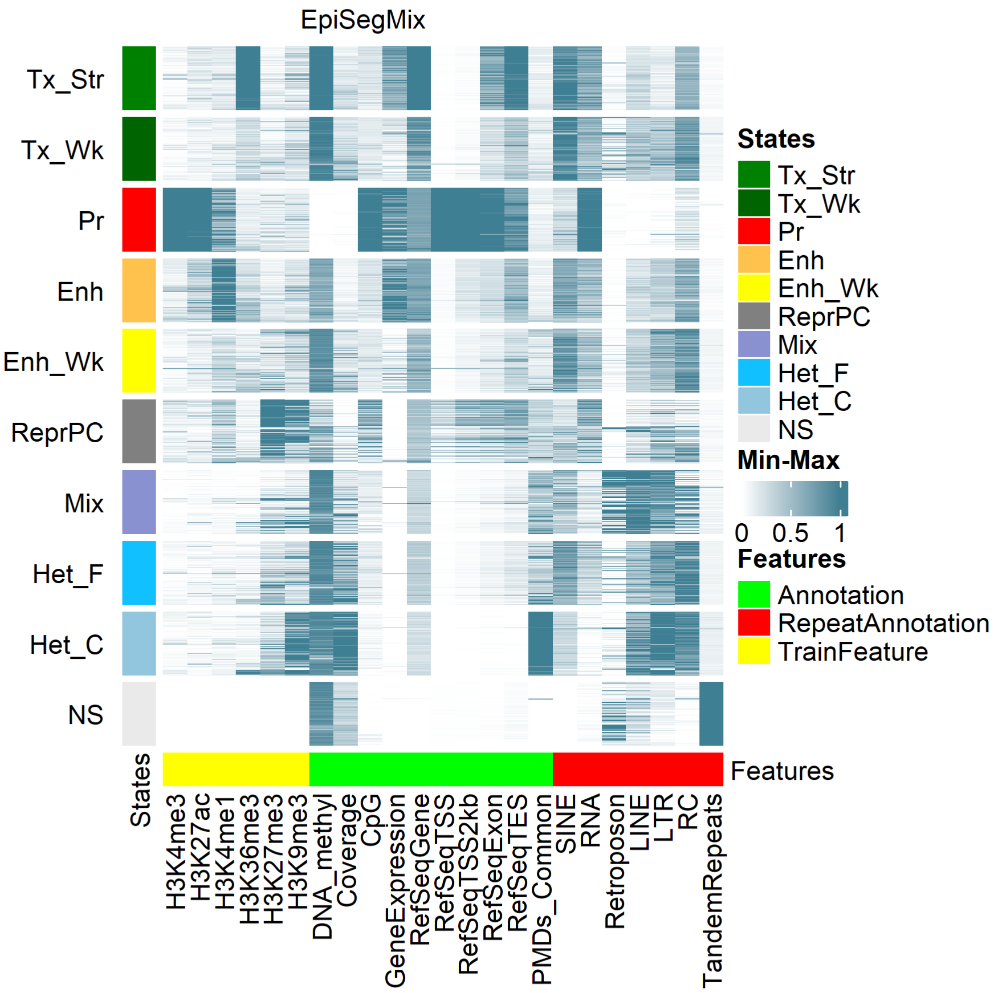

Figure S5 EpiSegMix states using six core histone marks annotated with biological functions. Labels are assigned based on the random forest classifier. Features on the x-axis are grouped into train (yellow), which are used in the model; annotation (green); and repeat annotation (red).

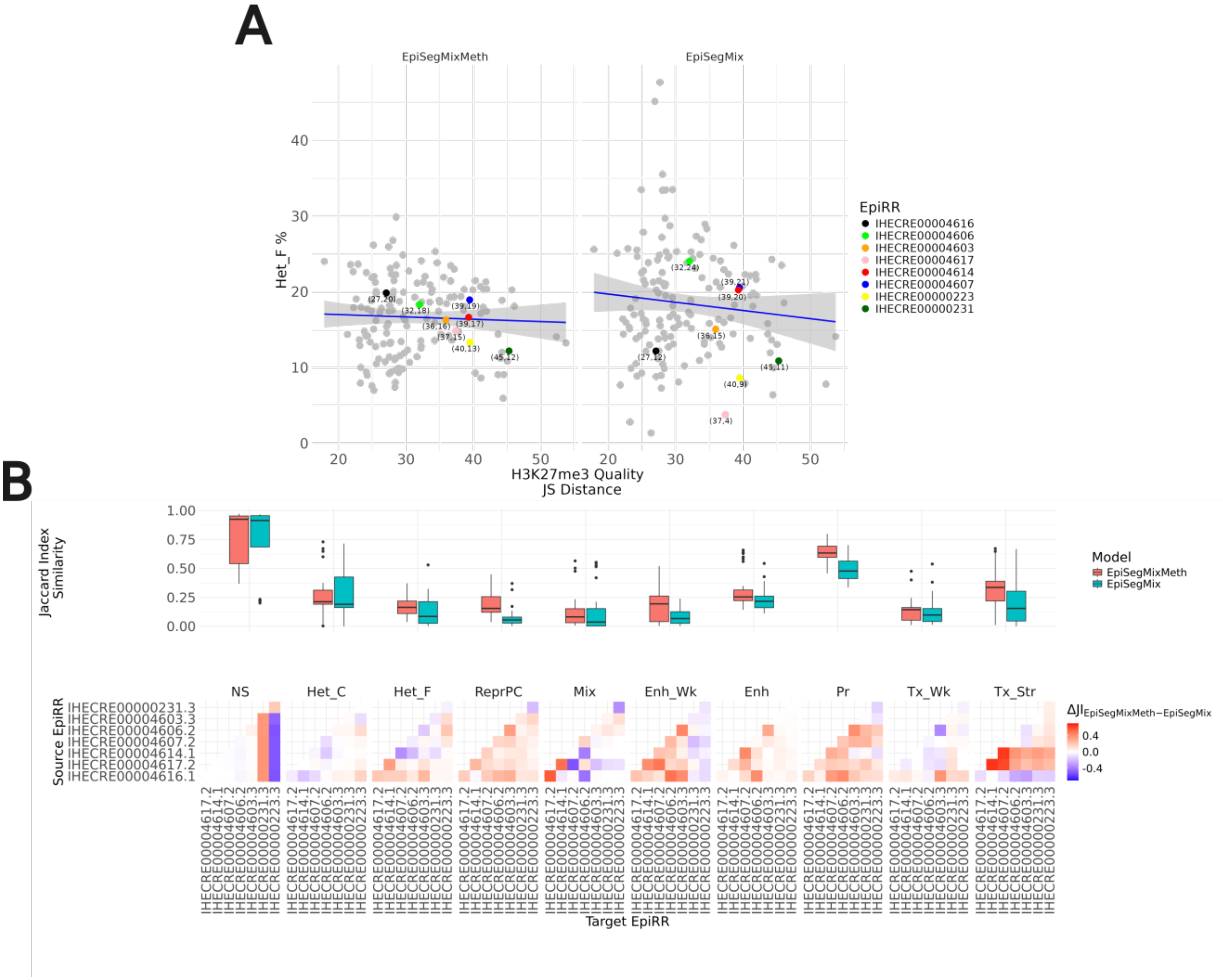

Figure S6 DNA methylation stabilizes segmentation calling in samples with reduced H3K27me3 ChIP signal quality : (A) *Left*: Eight samples from the same cell type, luminal epithelial, showing the quality of histone mark H3K27me3 on the x-axis, measured by synthetic Jenson-Shannon distance with samples ordered in increasing quality of H3K27me3 and genomic coverage of facultative heterochromatin (Het_F) defined by EpiSegMixMeth ESMM on the y-axis as expected effect state by H3K27me3. The smooth linear regression fit between the Het_F state coverage and quality of H3K27me3, shown in blue, with variance. *Right*: Same as *Left*, for states defined using EpiSegMix ESM. (B) *Up*: Boxplot comparing the ESMM and ESM models for all luminal cell samples per state (x-axis), assessing robustness using the Jaccard index (y-axis). High scores indicate that the genomic regions annotated are consistent across samples for that model. *Bottom*: Heatmap comparing EpiSegMixMeth (ESMM) and EpiSegMix (ESM) performance per sample across all 10 epigenetic states. Red/blue denotes relative ESMM out- or underperformance, respectively, against ESM by annotating consistency of regions (Jaccard Index); white indicates comparable performance.

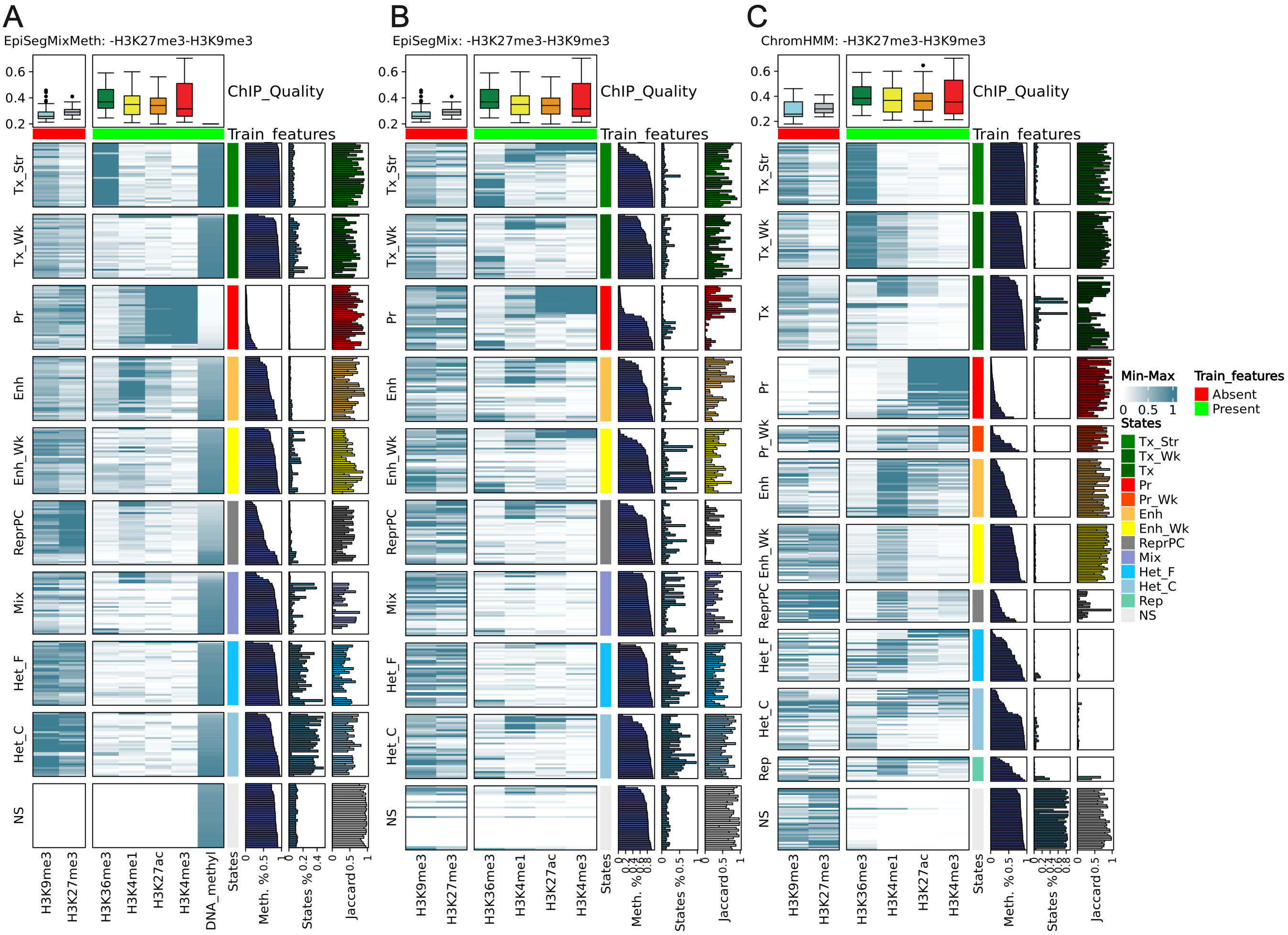

Figure S7 ESMM with DNA methylation (A) supports a range of regulatory and broad state recoveries in epigenomes lacking H3K27me3 and H3K9me3. The figure shows a comparison to chromatin-only-based models (B), EpiSegMix, and (C) ChromHMM. Composition of each subfigure: Heatmap displaying the enrichment of present histone marks (green bar) plus DNA methylation (only for ESMM) and absent heterochromatic histone marks (red bar). Each row represents one of the 40 T- or B-cell epigenome samples, and rows are grouped into states to enhance state-specific enrichments. Emissions within each state are ordered in relation to the increasing average DNA methylation per state. Box plots above the heatmaps display the synthetic Jensen-Shannon distance of histone marks across these samples as quality metrics. On the right, we additionally show the following annotations. Left: Average methylation per state for each sample. Middle: Genomic coverage of the state within the sample. Right: Similarity score (Jaccard index) measured between the state in the reduced model and the corresponding state in the complete model defined by core histone marks and DNA methylation for each sample.

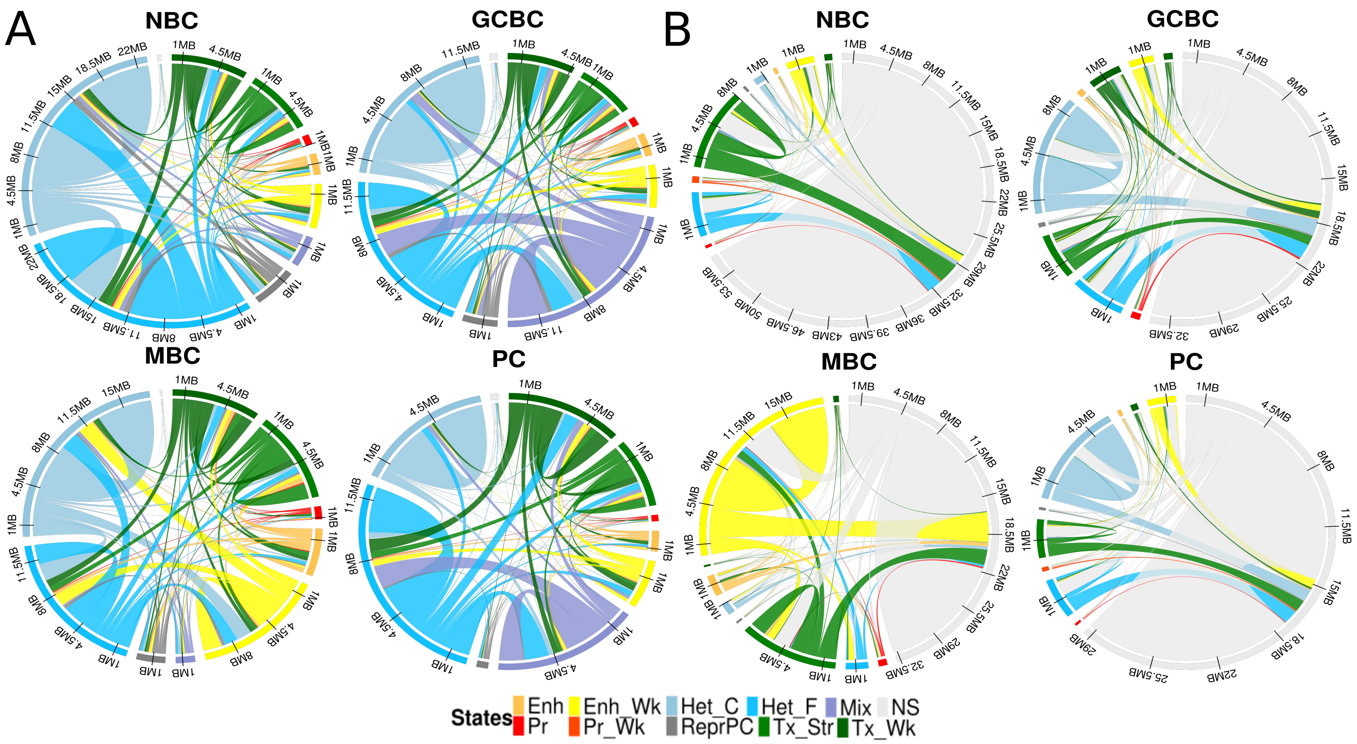

Figure S8 3D contacts in various B-cells in relation to ESMM(A) and, ChromHMM (B) segmentation: (A) The coloring highlights the inter- and intra-state 3D contacts in relation to ESMM segmentation. Contacts are derived from normalized Hi-C data with a maximum distance of 4 MB between positions. The cell-specific Circos plots summarize the contacts between and within states for naive B-cells (NBC), germinal center B-cells (GCBC), memory B-cells (MBC), and plasma cells (PC). (B) Same as (A), but for states defined by ChromHMM.

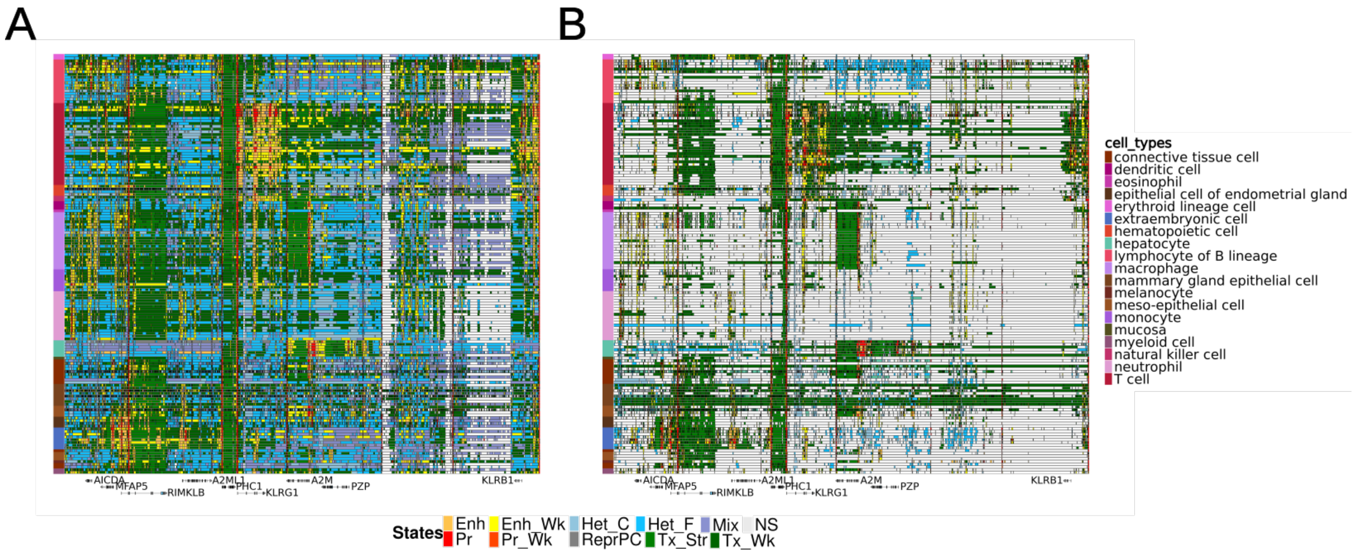

Figure S9 Comparison of ESMM and ChromHMM segmentation patterns ordered by cell type: (A) EpiSegMixMeth segmentation tracks as shown in Fig. 1 D (chr12:8550000-9650000) (B) ChromHMM segmentation tracks across this region grouped in the same order as (A). Note the extensive non-signal and Het-C and Het-F states in (B) particularly for monocyte and macrophage samples.

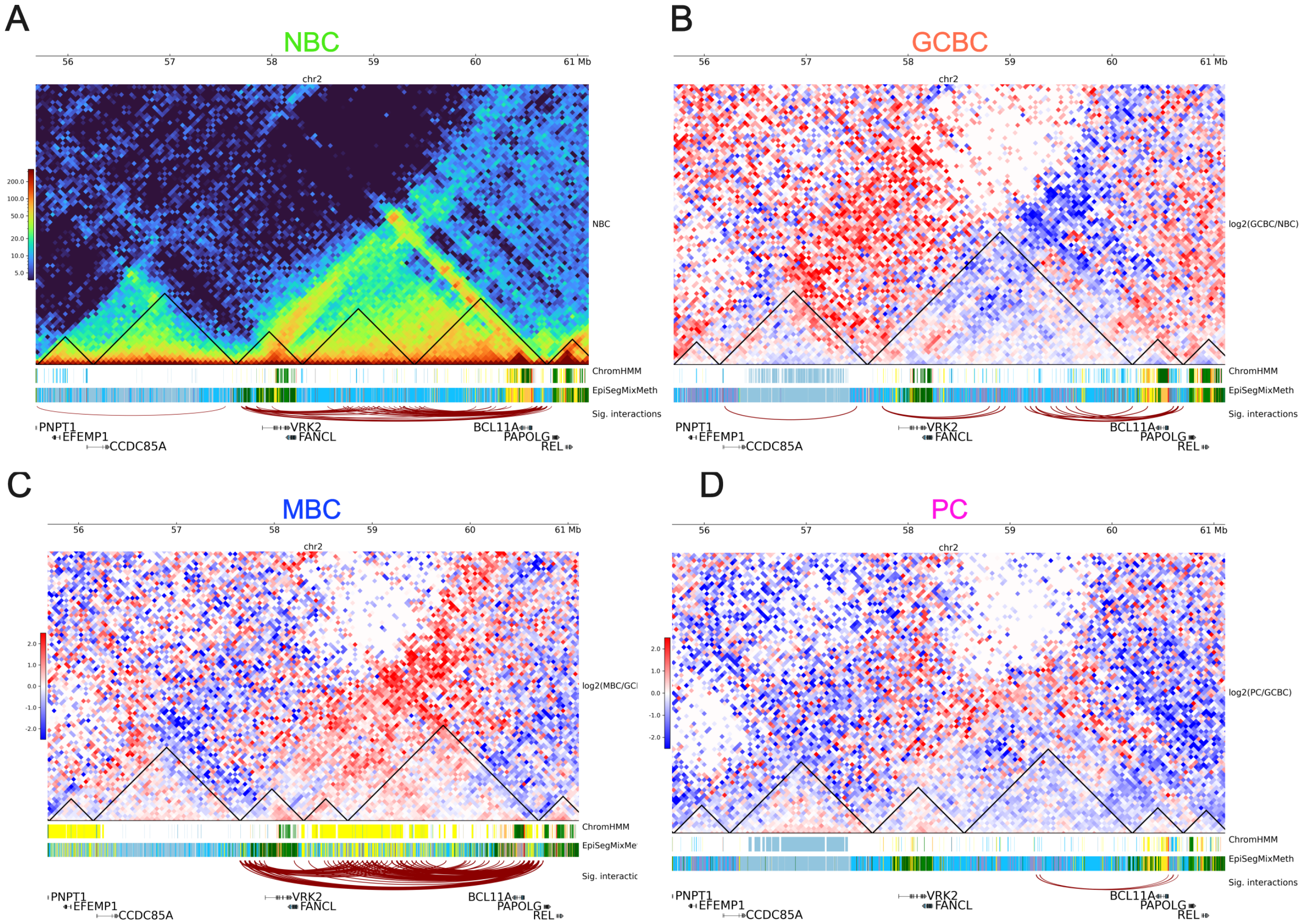

Figure S10 B-cell differentiation state transition. (A) Sequentially from top to bottom: Hi-C contact maps of NBC with TAD calls (black triangles), ChromHMM segmentation followed by EpiSegMixMeth segmentation, and significant 1MB interactions (thickness signifies importance). (B) Similar display as (A) for GCBC, except Hi-C maps show log fold changes from Naive B-cells, with red indicating increased interactions and blue indicating reductions. (C, D) As per (B) for MBC and PC, respectively, changes are relative to GCBC.

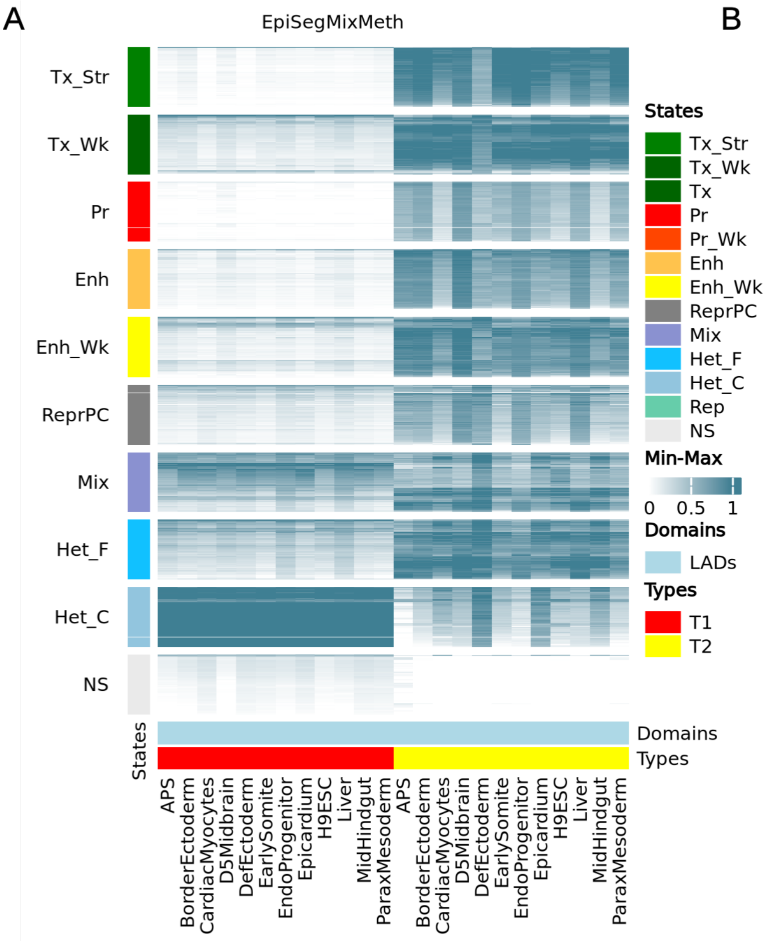

B

Feature	T1-LADs	T2-LADs
Defining mark	LAMIN B1 occupancy	LAMIN B1 occupancy
Median domain size	~290 kb	~280 kb
Genome coverage	~32%	~35%
LB1 enrichment	High	Intermediate
H3K9me2 enrichment	High	Intermediate
H3K9me3 & H3K27me3	High	Intermediate
Replication timing	Constitutive late	Switch domains
GC content	Lowest	Intermediate
B compartment	Enriched	Enriched
Gene density	Low	Intermediate
Gene expression	Low	Intermediate
Position relative to lamina	Embedded within	Within & adjacent
Cell-type variability	Stable (constitutive-like)	Dynamic (facultative)
Transposable elements	ERVL-MaLR enriched	L1, ERVL-MaLR enriched Divergent TE profile
Chromatin accessibility (ATAC-seq)	Low	Intermediate
Primary LAD+KDD overlap	T1-KDDs	T2-KDDs

Figure S11 Association between ESMM states and Lamina Associated Domains (LADs): ESMM states defined using six core histone marks and DNA methylation annotated as defined by Figure 2A. Features on the x-axis are grouped into repressive T1 (red) and intermediate T2 (yellow) LADs defined for 12 cell types in [55]. (B) Table summarizing various features from [55] for T2 and T1 LADs. T2 shows more the intermediate property between active and inactive regions whereas, T1 LADs are constant repressive regions.

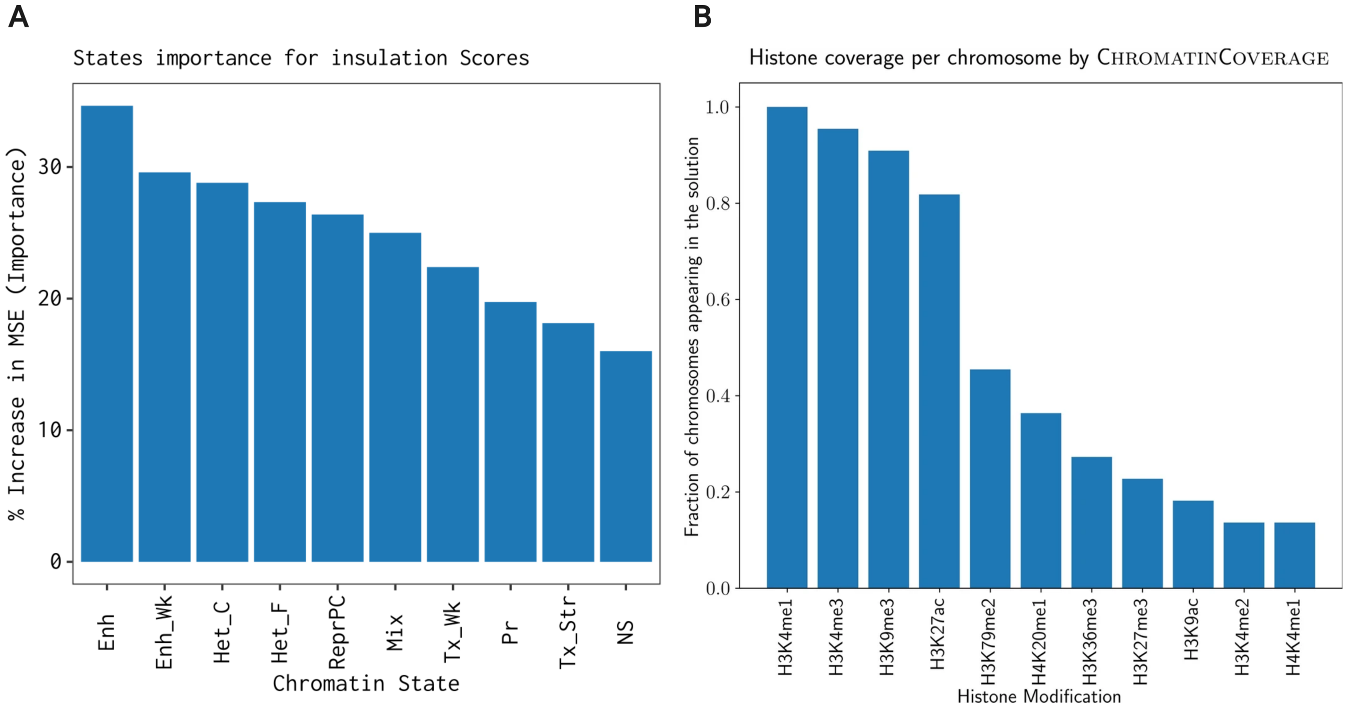

Figure S12 3D genome organization and ESMM-based chromatin states. (A) ESMM states on the X-axis and their importance on the Y-axis for predicting insulation scores, an essential metric for defining the boundaries of Topologically Associated Domains (TADs). Regulatory states, including enhancer (Enh, Enh_Wk) and heterochromatin (Het_C, Het_F), emerged as the most predictive features for TAD boundaries. (B) Adapted from [56, 57], showing the contribution (Y-axis) of each histone mark (X-axis) to the prediction of Hi-C interactions across species. Regulatory marks H3K4me1, H3K27ac (enhancer), and H3K4me3 (promoter), along with the heterochromatin mark H3K9me3, show the highest importance for Hi-C interactions, revealing substantial overlap with our findings.
